# CDK13 drives clear cell renal carcinoma through METTL16-mediated m^6^A modification of ACLY mRNA

**DOI:** 10.1038/s12276-025-01634-7

**Published:** 2026-02-12

**Authors:** Jinsuo Chen, Huan Liu, Yong Zhang, Junfei Gu, Xiaoli Wu, Fan Xuan, Changbao Qu, Hao Sun, Nanxi Yue, Chenxiao Yang, Hongye Zhao, Wenzeng Yang, Zhan Yang

**Affiliations:** 1 Clinical Medical College, Hebei University, Department of Urology, Affiliated Hospital of Hebei University, Baoding, China; 2 Department of Radiotherapy, The First Central Hospital of Baoding, Baoding, China; 3 Department of Urology, National Cancer Center/National Clinical Research Center for Cancer/Cancer Hospital, Chinese Academy of Medical Sciences and Peking Union Medical College, Beijing, China; 4 Department of Urology, The Second Hospital of Hebei Medical University, Shijiazhuang, China; 5 Department of Pediatrics Hematology-Oncology, The Second Hospital of Hebei Medical University, Shijiazhuang, China; 6 Department of Urology, Shijiazhuang People’s Hospital, Shijiazhuang, China; 7 Department of Biochemistry and Molecular Biology, The Key Laboratory of Neural and Vascular Biology, Ministry of Education of China, Hebei Medical University, Shijiazhuang, China; 8 Center of Tumor Immunology and Cytotherapy, Medical Research Center, The Affiliated Hospital of Qingdao University, Qingdao, China

**Keywords:** Renal cell carcinoma, Cancer metabolism, Phosphorylation, RNA modification

## Abstract

Cyclin-dependent kinase 13 (CDK13) has emerged as a critical regulator of oncogenic metabolism, but its role in rewiring lipid metabolism in clear cell renal cell carcinoma (ccRCC) remains undefined. Here we identify CDK13 as a master orchestrator of lipid dysregulation in ccRCC, demonstrating that it drives de novo lipogenesis through a phosphorylation-dependent RNA *N*^6^-methyladenosine (m^6^A) modification axis. Clinically, CDK13 overexpression correlates with advanced tumor stage, poor prognosis and aberrant lipid accumulation in patient-derived ccRCC tissues. Mechanistically, CDK13 directly phosphorylates the methyltransferase-like protein 16 (METTL16) at Ser329, augmenting its catalytic activity to install m^6^A modifications on ATP-citrate synthase (ACLY) messenger RNA. These m^6^A marks are selectively recognized by the YTHDC2 reader protein, leading to mRNA stabilization and increased acetyl-CoA production, which fuels lipogenesis and sustains ccRCC aggressiveness. Genetic or pharmacological disruption of the CDK13–METTL16–ACLY axis synergistically suppresses lipid deposition, tumor growth and metastasis in vitro and in vivo. Notably, targeting CDK13 with the small-molecule inhibitor 1NM-PP1 potentiates METTL16 depletion-mediated anticancer effects. Our findings establish a kinase-RNA modifier axis that links CDK13 to epitranscriptomic control of lipid metabolism, positioning the CDK13–METTL16–ACLY pathway as a promising target for precision therapies against ccRCC.

## Targeting CDK13 offers new hope for ccRCC treatment

Clear cell renal cell carcinoma (ccRCC) is a common type of kidney cancer known for abnormal fat buildup in cells. This study explores how a protein called cyclin-dependent kinase 13 (CDK13) might influence this process. Researchers found that CDK13 is often present in high amounts in ccRCC and is linked to worse outcomes for patients. The study involves analyzing cancer cells and tissues from patients, as well as using mouse models to understand the role of CDK13. They discovered that CDK13 helps increase the production of fats in cancer cells by affecting another protein, METTL16, which modifies RNA. This modification helps stabilize the RNA of a key enzyme, ACLY, which is crucial for fat production. Findings suggest that targeting the CDK13–METTL16–ACLY pathway could be a new way to treat ccRCC by disrupting the cancer’s ability to produce fats.

This summary was initially drafted using artificial intelligence, then revised and fact-checked by the author.

## Introduction

Clear cell renal cell carcinoma (ccRCC), the predominant subtype of renal cancer, is pathognomonically characterized by metabolic reprogramming, particularly aberrant lipid accumulation^1–3^. Tumor cells exploit de novo lipogenesis to sustain rapid proliferation, membrane biogenesis and survival under nutrient-deprived microenvironments^4^. Despite therapeutic advances, ccRCC remains lethal in metastatic stages due to intrinsic resistance to conventional therapies^5,6^. A hallmark of ccRCC is the ectopic lipid deposition driven by dysregulated expression of lipogenic enzymes such as ATP-citrate lyase (ACLY) and fatty acid synthase (FASN)^7,8^. Although lipid metabolism is recognized as a cornerstone of ccRCC pathogenesis, the molecular mechanisms underlying its dysregulation—particularly the interplay between oncogenic signaling and post-transcriptional RNA modifications—remain poorly understood.

ACLY, the rate-limiting enzyme converting citrate to acetyl-CoA, bridges glycolysis and lipid synthesis by providing substrates for fatty acid and cholesterol biosynthesis^9,10^. Its overexpression is a hallmark of multiple cancers, including ccRCC, where it fuels aggressive phenotypes and therapy resistance^11–13^. Although transcriptional and post-translational regulation of ACLY are partially characterized^14^, the role of RNA modifications in stabilizing ACLY messenger RNA remains unknown. Recent studies implicate *N*^6^-methyladenosine (m^6^A), the most abundant RNA modification, in regulating mRNA stability and translation^15^. However, whether m^6^A writers, readers or cyclin-dependent kinase (CDK)-driven phosphorylation modulate ACLY expression in ccRCC is unclear.

CDKs, traditionally known for cell cycle regulation, are increasingly implicated in metabolic reprogramming^16,17^. Cyclin-dependent kinase 13 (CDK13), a transcriptional CDK, phosphorylates RNA polymerase II to regulate RNA splicing and transcriptional elongation^18^. Our prior work revealed the role of CDK13 in prostate cancer, where it promotes lipid deposition via NSUN5-mediated m^5^C modification of ACC1 mRNA^19^. In addition, CDK13 forms a feedback loop with circCDK13 and E2F5 to drive prostate carcinogenesis^20^. Despite these advances, CDK13’s function in ccRCC—a malignancy distinct in its metabolic dependencies—remains unexplored. Given ccRCC’s reliance on lipid anabolism, we hypothesized that CDK13 might orchestrate lipid metabolic rewiring through novel epitranscriptomic mechanisms.

Here, we uncover a CDK13–METTL16–ACLY axis that drives lipid metabolic reprogramming in ccRCC. We demonstrate that CDK13 phosphorylates METTL16 to enhance m^6^A deposition on ACLY mRNA, which is stabilized by YTHDC2, thereby amplifying lipogenesis and tumor progression. This study bridges CDK signaling with RNA epitranscriptomics, revealing a novel mechanism by which metabolic enzymes are post-transcriptionally regulated in cancer.

## Materials and methods

### Cell culture and transfection

The human renal carcinoma cell lines 786-O (TCH-C107; HyCyte) and 769-P (TCH-C106; HyCyte) were maintained in RPMI-1640 medium (Gibco) supplemented with 10% heat-inactivated fetal bovine serum (Clark Bioscience), 100 U/mL penicillin and 100 μg/mL streptomycin under standard culture conditions. Cells were transiently transfected using Lipofectamine 2000 reagent, following established protocols^20,21^. Gene-specific siRNAs and scrambled control duplexes were synthesized by GenePharma.

### Clinical specimens and ethical compliance

Tumor and adjacent normal tissues were collected from treatment-naive patients with ccRCC during surgery. All cases were histologically confirmed and without prior therapy. This study was approved by the Institutional Review Board of the National Cancer Center/Cancer Hospital, CAMS (approval no. NCC2024C-307), and verbal consent was obtained from all participants in accordance with the Declaration of Helsinki.

### Lentiviral vector generation

Lentiviral constructs were generated following previously established protocols^19,22^. Lentiviral overexpression vectors (oeCon, oeCDK13, oeACLY and oeMETTL16) were packaged in HEK-293T cells using calcium phosphate transfection with psPAX2 and pMD2.G plasmids. Viral supernatants were concentrated by PEG precipitation and ultracentrifugation. Short hairpin RNA (shRNA) lentiviral particles were purchased from GeneChem.

### Luciferase reporter analysis

The ACLY CDS and 3′ untranslated region (3′UTR) were cloned into pGL3-Control vectors. Mutant reporters with A-to-T substitutions at key m6A motifs (nucleotides 3435, 3638) were generated. 786-O cells were cotransfected with reporter and pRL-TK plasmids^23^. Luciferase activity was measured 24 h post transfection and normalized to Renilla luminescence.

### Cell proliferation assessment

Proliferation was evaluated using CCK-8, clonogenic and EdU assays. For CCK-8, the cells seeded in 96-well plates were cultured for 24–72 h and incubated with CCK-8 solution for 4 h, and the absorbance was measured at 495 nm. For the clonogenic assay, the cells (100 cells per well) were plated in six-well plates. After 14 days, colonies were fixed, stained with crystal violet and counted. For the EdU assay, the cells were incubated with 10 μM EdU for 4 h, fixed, permeabilized and stained with Alexa Fluor 555 and Hoechst 33342. The EdU-positive rate was calculated using ImageJ.

### Histopathological and immunofluorescence evaluation

ccRCC and matched normal tissues were fixed, paraffin-embedded and sectioned for H&E staining and immunofluorescence following established protocols^19^. For immunofluorescence, the samples were permeabilized, blocked and incubated with primary antibodies at 4 °C overnight, followed by species-matched fluorescent secondary antibodies and 4,6-diamidino-2-phenylindole (DAPI) counterstaining. The images were acquired using a Leica DM6000B system and analyzed with LAS X software.

### Western blot analysis

Total protein was extracted with RIPA buffer, quantified by bicinchoninic acid assay and separated by SDS–polyacrylamide gel electrophoresis^19,24^. Proteins were transferred to polyvinylidene fluoride membranes, blocked and incubated overnight at 4 °C with primary antibodies: CDK13 (1:1000, DF10026), ACLY (1:1000, 15421-1-AP), ACACA (1:1000, 21923-1-AP), FASN (1:500, 10624-2-AP), SCD1 (1:2000, 28678-1-AP), METTL16 (1:2000, 19924-1-AP), Flag (1:1000, 20543-1-AP), YTHDC2 (1:1000, 27779-1-AP), β-actin (1:20000, 66009-1-Ig) and phospho-(Ser) CDK substrate antibody (1:500, #2324). After incubation with HRP-conjugated secondary antibodies, signals were detected using chemiluminescent substrate.

### Real-time qPCR

Total RNA was extracted from cells and tissues and reverse transcribed into complementary DNA using SuperScript III Reverse Transcriptase. Quantitative PCR (qPCR) was performed on a CFX96 system with SYBR Green Master Mix. Relative mRNA expression levels were calculated by the 2^−ΔΔCt^ method, normalized to GAPDH. Primer sequences are provided in Supplementary Table 3.

### Co-immunoprecipitation assay

Protein interactions were analyzed by co-immunoprecipitation. The cultured cells were lysed, and the supernatants were incubated with specific antibodies (anti-CDK13, anti-METTL16 or anti-Flag) at 25 °C for 1 h. Protein A/G magnetic beads were then added and incubated for another hour. After washing, the bound proteins were eluted and subjected to western blot analysis.

### Mouse xenograft tumorexperiment

All animal procedures were approved by the Institutional Animal Care Committee (NCC2024A243). Lentiviral-transduced 786-O cells with stable knockdown of CDK13, ACLY, CDK13/ACLY or METTL16 (5 × 10^6^ cells per mouse) were suspended in a 1:1 PBS/Matrigel mixture and implanted subcutaneously into 4-week-old male BALB/c nude mice (*n* = 8 per group). The inhibitor 1NM-PP1 (20 mg/kg) was administered intraperitoneally every 48 h from day 7 post implantation. Tumor volume was monitored twice weekly and calculated using the formula (length × width²)/2. After 28 days, mice were killed, and tumors were collected for molecular and histopathological analysis.

### ORO staining

Cultured cells or tissue cryosections (8 μm) were fixed with 4% formalin and stained using a commercial Oil Red O (ORO) kit (Solarbio) according to the manufacturer’s instructions. After mounting, images were captured with a Leica confocal microscope. The ORO-positive area was quantified using ImageJ and normalized to the number of DAPI-stained nuclei.

### Nile Red staining and BODIPY 493/503 staining

The 786-O and 769-P cells were fixed with 4% paraformaldehyde and stained using either a Nile Red lipid staining kit (Solarbio) or BODIPY 493/503 (Beyotime), following the manufacturers’ protocols. Images were acquired with a Leica confocal microscope, and the average fluorescence intensity was quantified using ImageJ and normalized to cell number.

### RNA immunoprecipitation assay

RNA–protein interactions were analyzed using the Dynabeads Protein G Kit. Cell lysates prepared in NETN buffer with inhibitors were incubated with antibody-conjugated magnetic beads at 4 °C for 2 h. After washing, bound RNA was purified, reverse transcribed into cDNA and analyzed by qPCR.

### MeRIP–qPCR

The m^6^A modifications on ACLY mRNA were analyzed using a commercial MeRIP kit. Fragmented RNA was immunoprecipitated with an m^6^A-specific antibody or control IgG antibody, coupled to magnetic beads. After incubation and washing, the enriched RNA was eluted and analyzed by RT–qPCR. Primers were designed based on predictions from the SRAMP database.

### RNA stability assays

786-O and 769-P cells were treated with 5 μg/mL actinomycin D to halt transcription. Total RNA was isolated at 0, 4, 8 and 12 h post treatment, followed by cDNA synthesis and SYBR Green-based qPCR. The mRNA half-life was calculated from the degradation curve using linear regression analysis.

### Statistical analysis

Data from three independent experiments are presented as mean ± s.e.m. Statistical significance between groups was determined by two-tailed Student’s *t*-test after confirming normality, with *P* < 0.05 considered significant. All analyses were performed using GraphPad Prism v9.0.

## Results

### CDK13 is overexpressed in ccRCC and correlates with poor prognosis

**Fig. 1:**
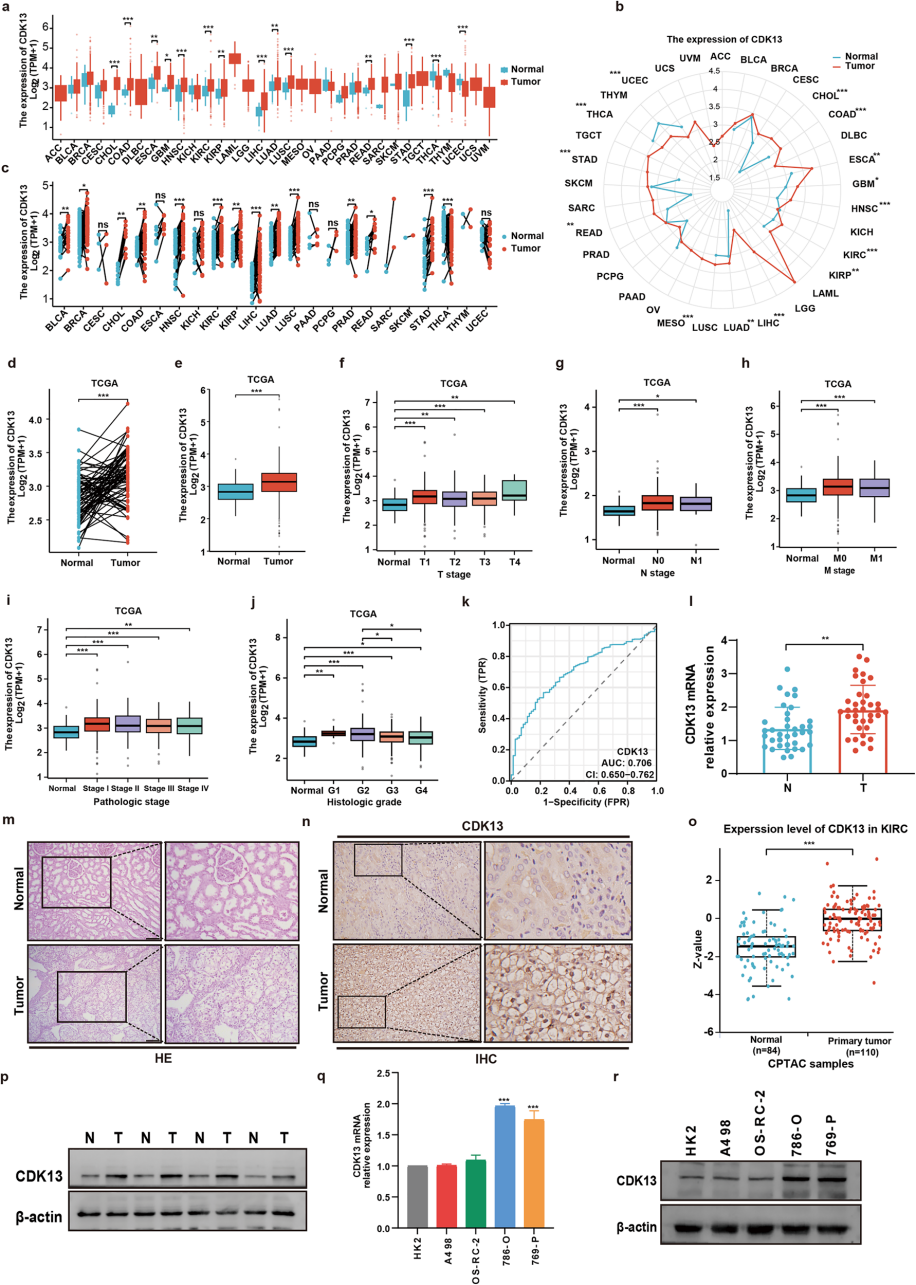
CDK13 is upregulated in ccRCC and correlates with unfavorable clinical outcomes. **a** Box plot showing pan-cancer analysis of CDK13 mRNA expression across 33 tumor types versus non-tumor tissues (TCGA, unpaired samples). **b** Radar chart visualizing the same CDK13 pan-cancer expression pattern as in (**a**). **c** Comparative analysis of CDK13 levels between TCGA tumor samples and matched normal tissues. **d**, **e** CDK13 mRNA expression in TCGA-KIRC tumors versus normal tissues. **d** Paired analysis. **e** Unpaired analysis. **f**–**j** Association of CDK13 mRNA levels with tumor size (**f**), lymph node metastasis (**g**), distant metastasis (**h**), pathologic stage (**i**) and histologic grade (**j**) in TCGA patients with ccRCC. **k** Diagnostic ROC curve evaluating CDK13’s predictive value for ccRCC in the TCGA cohort. **l** RT–qPCR analysis of CDK13 mRNA in 35 paired ccRCC tumors and adjacent normal renal tissues (**P < 0.01, paired *t* test). **m** Hematoxylin and eosin (HE) staining of normal renal tissues and ccRCC specimens. **n** Immunohistochemical (IHC) staining of CDK13 in normal and ccRCC tissues. Scale bar, 100 μm. **o** CDK13 protein expression in KIRC tumors versus normal tissues from the CPTAC database. **p** Western blot analysis of CDK13 protein in four paired ccRCC tumors (T) and adjacent normal tissues (N). **q**, **r** CDK13 mRNA (RT–qPCR) (**q**) and protein (western blot) (**r**) levels in human renal tubular epithelial cells (HK-2) and ccRCC cell lines (A498, 786-O, OS-RC-2 and 769-P). Elevated CDK13 expression was observed in 786-O and 769-P cells (***P < 0.001 versus HK-2; one-way analysis of variance). (*P < 0.05, **P < 0.01, ***P < 0.001; ns, not significant).

Our previous study found that upregulation of CDK13 promoted prostate cancer progression^20^. However, the expression and function of CDK13 in ccRCC remain unclear. Analysis of The Cancer Genome Atlas (TCGA) pan-cancer data revealed CDK13 overexpression across multiple malignancies, including kidney renal clear cell carcinoma (KIRC), also named ccRCC (Fig. 1a–c). In the TCGA-KIRC cohort, CDK13 mRNA levels were marked elevated in tumor tissues compared with adjacent normal tissues (Fig. 1d,e). Multiomics analysis of TCGA data revealed strong associations between CDK13 expression levels and clinicopathological features in ccRCC. As illustrated in Fig. 1f–j, elevated CDK13 expression exhibited strong positive correlations with tumor size, lymphatic node metastasis, distant metastasis, American Joint Committee on Cancer (AJCC) pathological staging and clinical grade classification. These findings collectively suggest that CDK13 may serve as a novel prognostic biomarker for KIRC progression. To evaluate the diagnostic utility of CDK13 in KIRC, receiver operating characteristic (ROC) curve analysis was performed. As depicted in Fig. 1k, CDK13 expression demonstrated notable discriminative capacity between tumor and normal tissues, yielding an area under the curve of 0.706 (95% confidence interval 0.650–0.762). This performance indicates CDK13 holds considerable promise as a non-invasive biomarker for KIRC diagnosis, with satisfactory sensitivity and specificity across tested cohorts.

To investigate the potential role of CDK13 in human ccRCC pathogenesis, we then examined its expression in clinical samples levels. The RT–qPCR results showed that ccRCC tissues exhibited markedly elevated CDK13 mRNA expression compared with normal controls (Fig. 1l). This finding was corroborated by immunohistochemical analysis (Fig. 1m,n), which demonstrated marked upregulation of CDK13 protein expression in tumor specimens. To expand our analysis, we performed large-scale proteomic evaluation using the CPTAC database. This revealed that CDK13 protein expression was strikingly higher in KIRC tissues (*n* = 110) versus normal renal tissues (Fig. 1o). Western blot analysis in the clinical sample further confirmed this differential expression pattern (Fig. 1p). For in vitro validation, we assessed CDK13 expression across four ccRCC cell lines (786-O, 769-P, A498 and OS-RC-2) using both RT–qPCR and western blotting. Our data revealed substantial upregulation of CDK13 mRNA and protein levels in 786-O and 769-P cells compared with the normal renal epithelial cell line HK-2 (Fig. 1q,r). Based on these findings, we selected 786-O and 769-P cells for subsequent functional studies. Collectively, these findings establish that CDK13 is universally upregulated in ccRCC at both the mRNA and protein levels, suggesting its potential involvement in oncogenic progression of this malignancy.

### CDK13 upregulation accelerates proliferation and lipid metabolic reprogramming in ccRCC cells

**Fig. 2:**
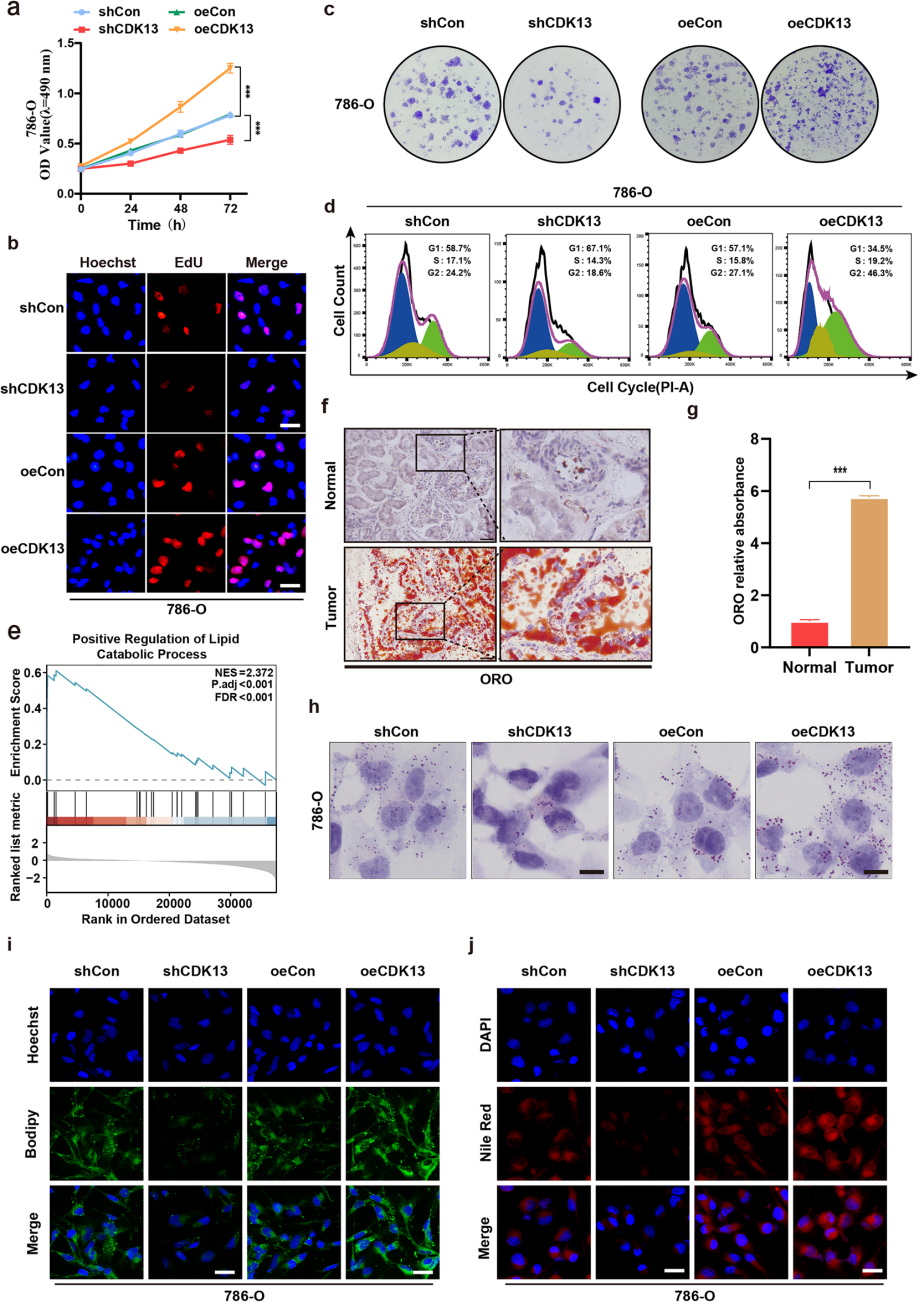
CDK13 enhances proliferation and drives fatty acid biosynthesis and lipid accumulation in ccRCC. **a** Cell viability assessed by CCK-8 assay in 786-O cells transfected with CDK13-overexpressing (oeCDK13) or CDK13-knockdown (shCDK13) vectors versus their respective controls. oeCon, empty overexpression vector; shCon, scramble shRNA control. **b** EdU incorporation assay evaluating proliferation in 786-O cell with CDK13 modulation. Scale bar, 50 μm. **c** 786-O cells were treated as in (**a**), and a colony formation assay was used to evaluate proliferation. **d** The cell cycle phase distribution analyzed by flow cytometry in a CDK13-manipulated 786-O cell. **e** A GSEA analysis of lipid catabolic pathways regulated by CDK13 in ccRCC (false discovery rate <0.001; ***P < 0.001). **f**, **g** ORO staining of lipid deposition in normal renal tissues and ccRCC specimens. **f** Representative images (scale bar, 100 μm). **g** Quantitative analysis of ORO staining intensity (***P < 0.001). **h** ORO staining of lipid accumulation in 786-O cell transfected with CDK13-overexpressing (oeCDK13), CDK13-knockdown (shCDK13) vectors or their respective controls. oeCon, empty vector; shCon, scramble shRNA. Scale bar, 25 μm. **i** BODIPY 493/503 staining of lipid droplets (LDs) in 786-O cell visualized by confocal microscopy. Scale bar, 50 μm. **j** Nile Red staining of neutral lipids in CDK13-modulated 786-O cell. Scale bar, 20 μm (*P < 0.05, **P < 0.01, ***P < 0.001 versus corresponding controls).

To elucidate the functional role of CDK13 in ccRCC progression, we conducted loss-of-function and gain-of-function studies to evaluate its impact on cellular proliferation. Knockdown of CDK13 using shRNA markedly suppressed proliferation in 786-O and 769-P cells, as evidenced by CCK-8 assays (Fig. 2a and Supplementary Fig. 1a). Conversely, CDK13 overexpression markedly enhanced cell growth in these models. These findings were corroborated by EdU incorporation and colony formation assays, which demonstrated that CDK13 depletion inhibited proliferation, while its ectopic expression promoted clonal expansion (Fig. 2b,c and Supplementary Fig. 1b–f). Flow cytometric analysis further revealed that CDK13 knockdown caused a marked reduction in G2-phase cells and concomitant increases in G1- and S-phase populations in both cell lines. Notably, CDK13 overexpression induced a reverse cellular cycle distribution, with a higher proportion of cells entering G2 phase (Fig. 2d and Supplementary Fig. 1g–i). Collectively, these data establish CDK13 as a critical regulator of cell cycle progression and proliferation in ccRCC.

Building upon our prior evidence linking CDK13 overexpression to tumorigenesis in prostate cancer^19^, we hypothesized that CDK13 might similarly regulate renal carcinogenesis through metabolic reprogramming. To test this hypothesis, we first performed gene set enrichment analysis (GSEA) on TCGA datasets and identified a strong positive correlation between CDK13 expression and lipid catabolic pathways in ccRCC (Fig. 2e). This was supported by histopathological evaluation showing marked lipid accumulation in primary ccRCC tissues compared with matched normal renal parenchyma, as assessed by ORO staining (Fig. 2f,g). To establish a causal relationship, we conducted loss-of-function and gain-of-function studies in ccRCC cell lines. CDK13 overexpression markedly increased intracellular lipid content, whereas shRNA-mediated CDK13 knockdown reduced lipid deposition in both 786-O and 769-P cells, as quantified by ORO staining (Fig. 2h and Supplementary Fig. 2a,b). Furthermore, BODIPY 493/503 staining revealed that CDK13 depletion decreased neutral lipid storage, while its overexpression enhanced lipid droplet formation (Fig. 2i and Supplementary Fig. 2c–e). Nile Red staining demonstrated that CDK13 modulated both triglyceride and cholesterol ester levels in a dose-dependent manner (Fig. 2j and Supplementary Fig. 2f–h). Collectively, these data establish CDK13 as a critical regulator of lipid metabolic reprogramming in ccRCC.

### CDK13 promotes lipid deposition through ACLY-mediated de novo lipogenesis

**Fig. 3:**
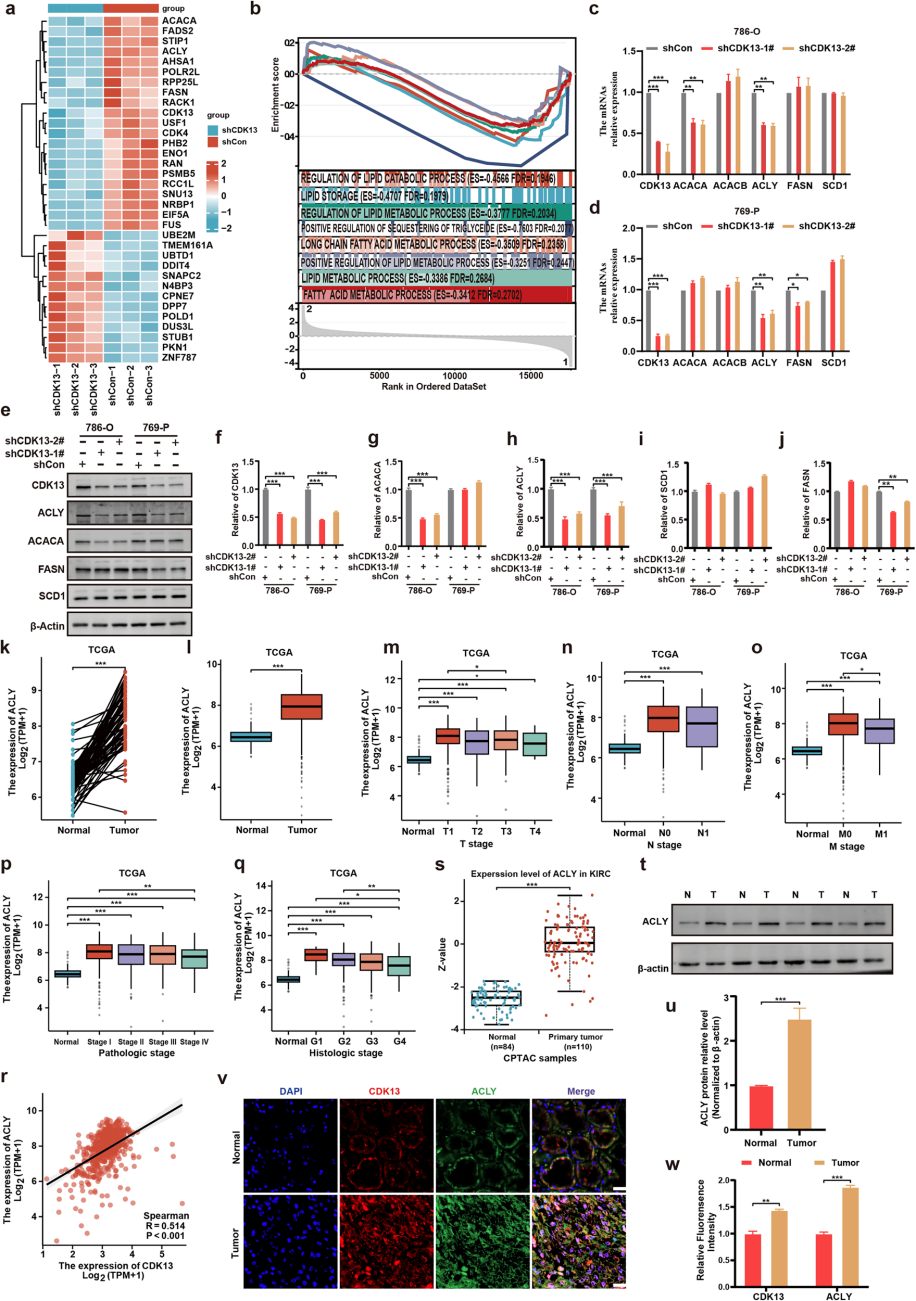
CDK13 positively correlates with ACLY expression in ccRCC. **a** Transcriptome sequencing analysis of 786-O cells with or without stable CDK13 knockdown (shCDK13 versus shCon; *n* = 3). The heat map depicts markedly altered lipid metabolism-associated molecules (upregulated (red), downregulated (blue)). **b** GSEA enrichment scores (ES) for lipid-related pathways: lipid storage (ES of 0.47, false discovery rate of 0.20) and regulation of lipid catabolic process (ES = 0.46, FDR = 0.19). **c**, **d** qRT–PCR analysis of fatty acid synthase (FASN, ACLY, ACACA) mRNA levels in CDK13-knockdown (shCDK13-1#, shCDK13-2#) 786-O and 769-P cells versus scramble shRNA controls (shCon). **c** Results in 786-O cells. **d** Results in 769-P cells. **e** Representative immunoblots of ACLY, FASN, and ACACA in 786-O and 769-P cells transduced with shCDK13-1#, shCDK13-2#, or scramble shRNA control (shCon). **f**–**j** Densitometric quantification of the protein levels shown in (**e**). **f** Protein levels of CDK13; **g** protein levels of ACACA; **h** protein levels of ACLY; **i** protein levels of SCD1; **j** protein levels of FASN. **k**, **l** ACLY mRNA expression in TCGA-KIRC tumors versus normal tissues. **k** Paired analysis. **l** Unpaired analysis. **m**–**q** Association of ACLY expression with tumor size (**m**), lymph node metastasis (**n**), distant metastasis (**o**), pathologic stage (**p**) and histologic grade (**q**) in TCGA patients with ccRCC. **r** Positive correlation between CDK13 and ACLY mRNA levels in TCGA ccRCC cohort (Pearson’s correlation analysis). **s** ACLY protein expression in KIRC tumors versus normal tissues from CPTAC database. **t**, **u** Western blot validation of ACLY protein levels in four paired ccRCC tumors (T) and adjacent normal tissues (N). **v** Immunofluorescence costaining of CDK13 (red) and ACLY (green) in normal renal tissues and ccRCC specimens. Nuclei counterstained with DAPI (blue). Scale bar, 10 μm. **w** A quantification of CDK13/ACLY colocalization fluorescence intensity. The data represent mean ± s.e.m. of three independent experiments (*P < 0.05, **P < 0.01, ***P < 0.001 versus shCon).

To identify the molecular mechanisms underlying CDK13’s role in ccRCC, we performed transcriptomic analysis of 786-O cells following CDK13 knockdown. This revealed concerted downregulation of key enzymes involved in de novo lipogenesis, including *ACACA*, *FASN* and *ACLY* (Fig. 3a and Supplementary Table 1). GSEA further supported this observation by demonstrating a strong positive correlation between CDK13 expression and metabolic pathways related to triglyceride biosynthesis and lipid metabolism (Fig. 3b), suggesting CDK13 may regulate lipid accumulation through transcriptional control of fatty acid synthesis genes. To determine which lipogenic enzymes are directly regulated by CDK13, we performed shRNA-mediated CDK13 knockdown (two independent constructs, shCDK13-1# and shCDK13-2#) in 786-O and 769-P cells. qPCR analysis showed that CDK13 depletion specifically reduced ACACA and ACLY mRNA levels in 786-O cells and reduced FASN and ACLY mRNA expression in 769-P cells, although ACACB and SCD1 expression remained largely unaffected (Fig. 3c,d). Western blot analysis confirmed these findings at the protein level: ACACA and ACLY were downregulated in 786-O cells, whereas FASN and ACLY were reduced in 769-P cells following CDK13 loss (Fig. 3e–j). To establish clinical relevance, we analyzed TCGA datasets and found that ACLY mRNA expression was consistently elevated in ccRCC tissues compared with normal renal parenchyma (Fig. 3k,l). Elevated ACLY levels correlated with advanced clinicopathological features, including larger tumor size, lymph node metastasis, distant metastasis, higher pathologic stage and clinical grade (Fig. 3m–q). Moreover, CDK13 expression exhibited a strong positive correlation with ACLY in the TCGA ccRCC cohort (Fig. 3r). Using CPTAC, we verified that ACLY protein expression was robustly upregulated in ccRCC tissues (*n* = 110) versus normal controls (*n* = 84) (Fig. 3s). Western blot analysis of clinical specimens further confirmed that ACLY protein levels were consistently increased in ccRCC relative to adjacent normal tissues (Fig. 3t,u). Finally, double immunofluorescence staining revealed that CDK13 and ACLY colocalized in ccRCC tissues, with high CDK13 expression being associated with elevated ACLY levels (Fig. 3v,w). These data collectively establish a CDK13–ACLY axis that drives de novo lipogenesis and contributes to lipid metabolic reprogramming in ccRCC.

### ACLY mediates CDK13-induced lipid metabolic reprogramming and ccRCC progression

**Fig. 4:**
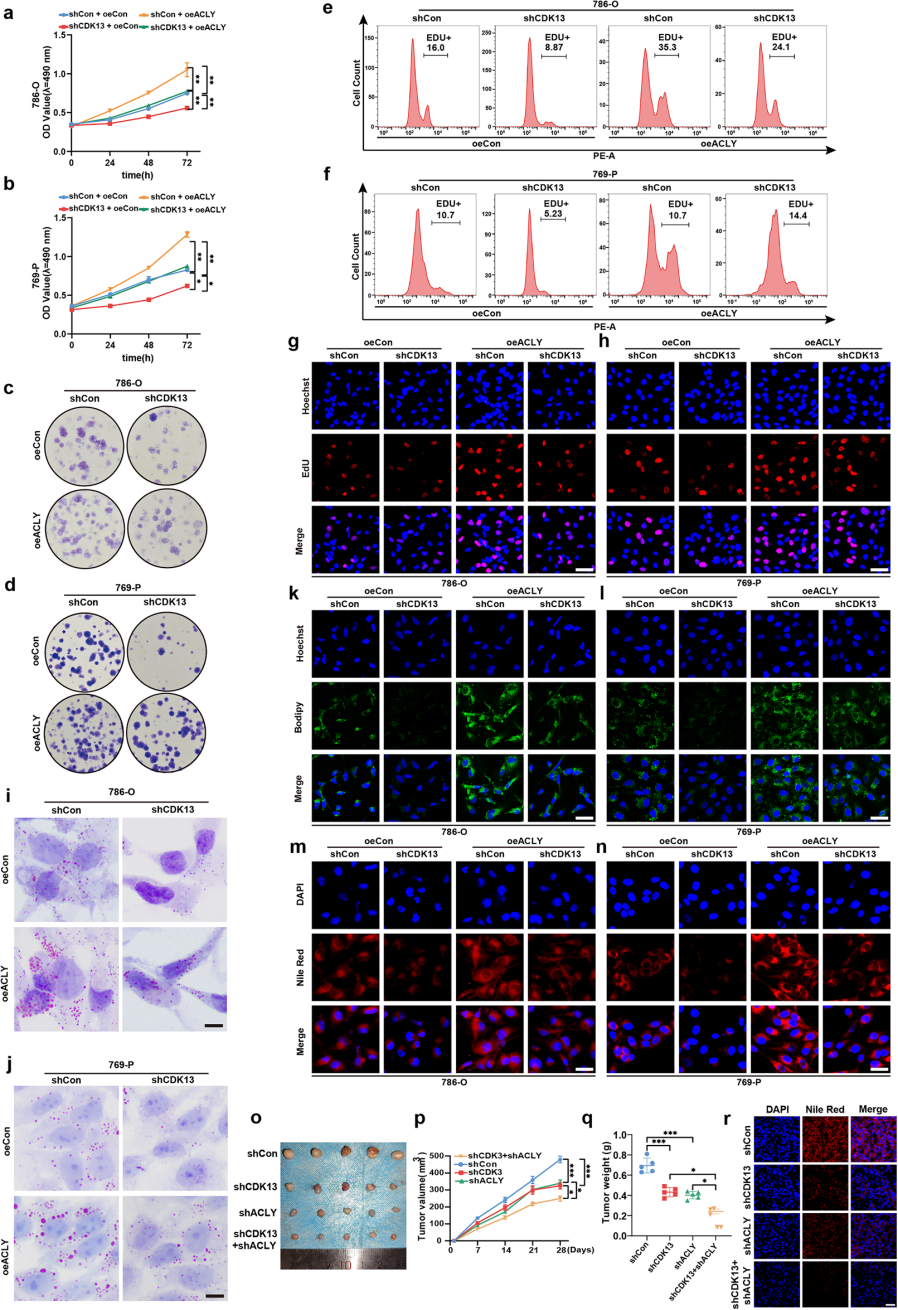
ACLY drives CDK13-mediated ccRCC progression and lipid deposition. **a**, **b** Cell viability assessed by CCK-8 assay in 786-O and 769-P cells transfected with CDK13-knockdown (shCDK13), ACLY-overexpressing (oeACLY) or combined vectors versus controls. shCon, scramble shRNA; oeCon, empty vector. **a** Results in 786-O cells. **b** Results in 769-P cells. **c**, **d** Colony formation assay evaluating proliferation in cells with CDK13/ACLY modulation. **c** Results in 786-O cells. **d** Results in 769-P cells. **e**, **f** EdU incorporation analysis by flow cytometry in CDK13-silenced and/or ACLY-overexpressing cells. **e** Results in 786-O cells. **f** Results in 769-P cells. **g**, **h** 786-O and 769-P cells were transfected as in (**a**, **b**), and an EdU assay was used to detect cellular proliferation. Scale bar, 50 μm. **g** Results in 786-O cells. **h** Results in 769-P cells. **i**, **j** ORO staining of lipid accumulation in 786-O and 769-P cells under CDK13/ACLY modulation. Scale bar, 25 μm. **i** Results in 786-O cells. **j** Results in 769-P cells. **k**–**n** Lipid droplet visualization via BODIPY 493/503 (green) and Nile Red (red) staining in CDK13/ACLY-manipulated cells. **k**, **l** BODIPY 493/503 staining (green). **k** 786-O cells. **l** 769-P cells. **m**, **n** Nile Red staining (red). **m** 786-O cells. **n** 769-P cells. Scale bar, 50 μm (applies to all images). **o** Subcutaneous xenograft tumor formation using 786-O cells with stable CDK13 knockdown (shCDK13), ACLY knockdown (shACLY) or dual knockdown (shCDK13 + shACLY). Representative tumor images (*n* = 5 per group). **p** Tumor volume calculated as (length × width^2^)/2 over 28 days. **q** Tumor weights post-resection at endpoint. **r** Nile Red staining of lipid accumulation in xenograft tumors. Scale bar, 50 μm. The data represent mean ± s.e.m. (*P < 0.05, **P < 0.01, ***P < 0.001 versus shCon).

To determine whether ACLY functions as a critical downstream effector of CDK13 in ccRCC, we performed complementary loss-of-function and gain-of-function studies. CDK13 knockdown potently inhibited cell viability and proliferation in 786-O and 769-P cells, while ACLY overexpression rescued this proliferative deficit (Fig. 4a,b). Moreover, ACLY overexpression not only restored clonal expansion capacity in both cell lines but also reversed the CDK13 depletion-induced growth arrest, as evidenced by colony formation assays (Fig. 4c,d). Flow cytometric analysis of EdU-labeled cells confirmed that ACLY overexpression accelerated S-phase entry and mitotic activity, thereby alleviating the CDK13-knockdown-associated G1/S-phase arrest (Fig. 4e,f). Similar results were observed in the EdU assay, as shown in Fig. 4g,h. Consistent with these findings, ACLY overexpression promoted lipid accumulation in both cell lines, as demonstrated by ORO, BODIPY 493/503 and Nile Red staining. Notably, ACLY overexpression partially restored lipid deposition levels in CDK13-depleted cells (Fig. 4i–n), establishing a functional link between CDK13, ACLY and lipid synthesis.

To validate the in vivo relevance, we generated orthotopic xenograft models using 786-O cells stably expressing CDK13 and/or ACLY shRNAs. Tumor growth analysis revealed that single-gene knockdown of CDK13 or ACLY potently reduced tumor volume (Fig. 4o,p) and weight (Fig. 4q) compared with control groups. Strikingly, dual knockdown of both genes exhibited synergistic growth inhibition (Fig. 4o–q), suggesting combinatorial targeting of this axis may hold therapeutic potential. Correspondingly, lipid content analysis by Nile Red staining demonstrated that genetic ablation of CDK13 or ACLY impaired lipid storage, while combined knockdown caused near-complete suppression of lipid accumulation (Fig. 4r). Collectively, these data establish ACLY as a critical mediator of CDK13-driven oncogenic lipid metabolism and ccRCC progression.

### CDK13 Iinteracts with and phosphorylates METTL16 to regulate ACLY expression

**Fig. 5:**
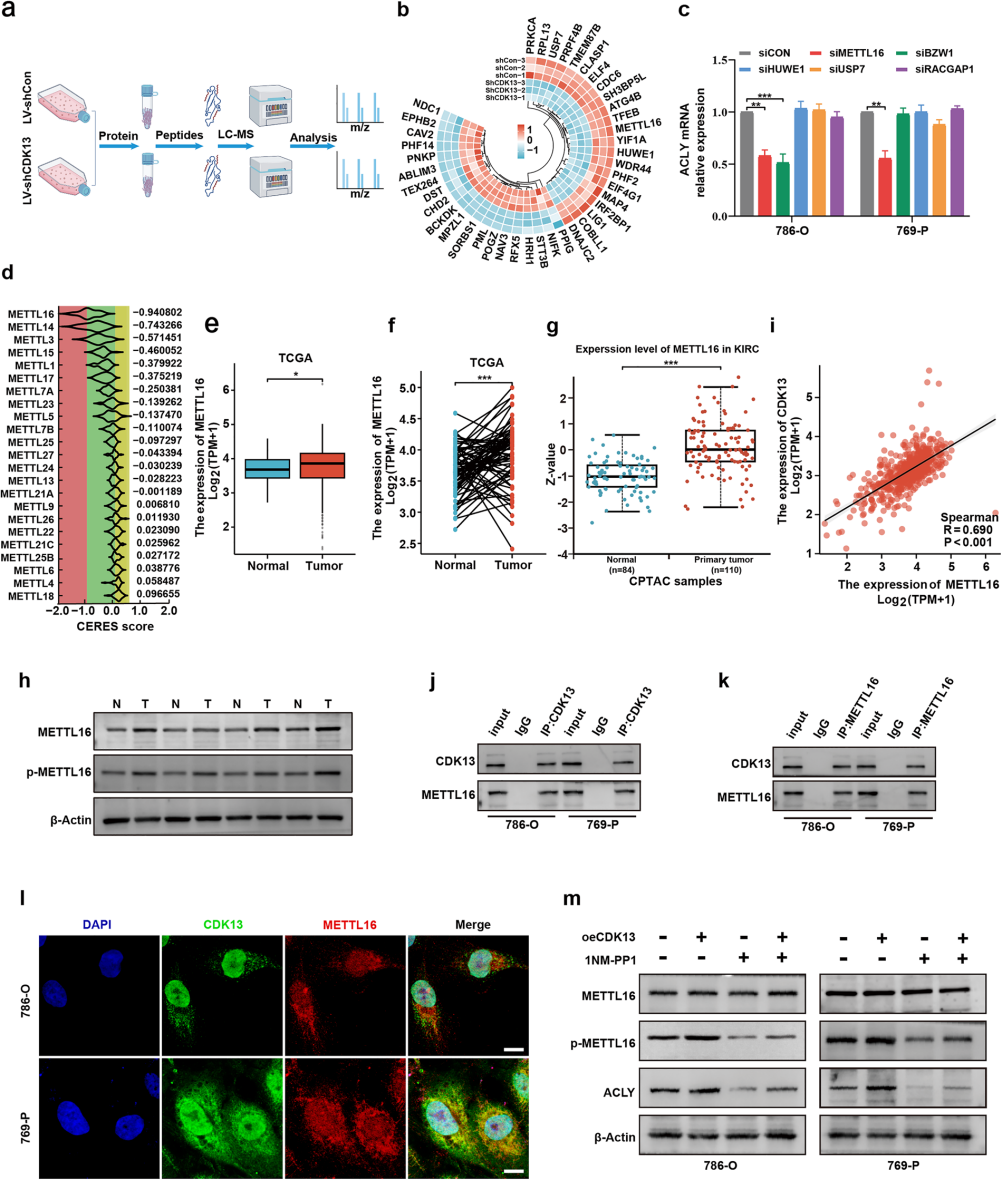
CDK13 regulates METTL16 interaction and phosphorylation in ccRCC. **a** A schematic of phosphoproteomic mass spectrometry workflow comparing 786-O cells with stable CDK13 knockdown (shCDK13) versus scramble control (shCon; *n* = 3). **b** A circular heat map depicting CDK13-associated phosphorylation changes (upregulated (red), downregulated (blue)). **c** ACLY mRNA levels measured by RT–qPCR in 786-O and 769-P cells following siRNA-mediated knockdown of METTL16, BZW1, HUWE1, USP7, or RACGAP1. **d** CRISPR–Cas9 screening of METTL family dependency in ccRCC using DepMap CERES scores (lower scores indicate stronger essentiality). Right: the bar plots show the average CERES scores for METTL members. **e**, **f** METTL16 mRNA expression in TCGA-KIRC tumors versus normal tissues. **e** Unpaired analysis. **f** Paired analysis (tumor vs. matched adjacent normal tissue). **g** METTL16 protein levels in KIRC tumors versus normal tissues from CPTAC database. **h** Western blot analysis of METTL16 and phosphorylated METTL16 (p-METTL16) in four paired ccRCC tumors (T) and adjacent normal tissues (N). **i** Positive correlation between CDK13 and METTL16 mRNA in TCGA ccRCC cohort (Pearson’s correlation). **j**, **k** Co-immunoprecipitation (Co-IP) analysis of the interaction between CDK13 and METTL16. **j** IP with anti-CDK13 antibody, followed by immunoblotting for METTL16, in 786-O and 769-P cells. **k** Reciprocal IP with anti-METTL16 antibody, followed by immunoblotting for CDK13, in the same cell lines. **l** Immunofluorescence colocalization of CDK13 (red) and METTL16 (green) with DAPI nuclear counterstain (blue). Scale bar, 10 μm. **m** Western blot analysis of METTL16, p-METTL16 and ACLY in CDK13-overexpressing (oeCDK13) cells treated with 1NM-PP1 (10 μM). The data represent three independent experiments (*P < 0.05, **P < 0.01, ***P < 0.001 versus their corresponding controls).

To identify direct CDK13 targets involved in ACLY regulation, we performed phosphoproteomic analysis comparing CDK13-knockdown 786-O cells with control cells (Fig. 5a). This revealed 22 markedly downregulated phosphoproteins in CDK13-deficient cells. A heat map visualization highlighted METTL16 as a top candidate, along with BZW1, HUWE1, USP7 and RACGAP1 (Fig. 5b and Supplementary Table 2). We subsequently knocked down these five genes and assessed ACLY expression. Only METTL16 depletion specifically reduced ACLY mRNA levels in both 786-O and 769-P cells (Fig. 5c and Supplementary Fig. 3). Large-scale CRISPR–Cas9 knockout screens further supported METTL16’s importance, revealing its critical function for ccRCC cell survival among METTL family members (Fig. 5d). Given the critical roles of canonical m^6^A writers METTL3 and METTL14 in ccRCC, we assessed their potential involvement in the CDK13–METTL16 axis. Analysis of TCGA data revealed that METTL14 and METTL3 expression was not elevated in ccRCC tumors compared with normal tissues (Supplementary Fig. 4a,b), consistent with its reported tumor suppressor function^25–28^. Importantly, siRNA-mediated knockdown of METTL14 did not alter ACLY expression in either control or CDK13-overexpressing cells (Supplementary Fig. 4c). Similarly, knockdown of METTL3, an oncoprotein in ccRCC^29,30^, had no effect on ACLY expression regardless of CDK13 status (Supplementary Fig. 4d). These data indicate that the regulation of ACLY by CDK13 is independent of METTL3 and METTL14, underscoring the specificity of the CDK13–METTL16 pathway. Bioinformatic analysis of TCGA datasets confirmed that METTL16 mRNA was upregulated in ccRCC tissues compared with normal renal parenchyma (Fig. 5e,f). Protein expression analysis using CPTAC demonstrated robust METTL16 upregulation in ccRCC samples (Fig. 5g). Western blot confirmed elevated METTL16 and p-METTL16 protein levels in clinical specimens (Fig. 5h). To establish a functional connection, we analyzed the TCGA-KIRC dataset and found a positive correlation between CDK13 and METTL16 expression (Fig. 5i). Co-immunoprecipitation assays revealed a direct physical interaction between CDK13 and METTL16 in ccRCC cells (Fig. 5j,k). Double immunofluorescence staining further confirmed their colocalization in cellular compartments (Fig. 5l). To determine whether CDK13 phosphorylates METTL16 to regulate ACLY, we treated CDK13-overexpressing cells with 1NM-PP1, a Src kinase inhibitor. While CDK13 overexpression increased phosphorylated METTL16 (p-METTL16) and ACLY levels, 1NM-PP1 treatment abolished this effect, partially reversing CDK13’s promotional impact on both molecules (Fig. 5m). These findings collectively establish a CDK13–METTL16–ACLY axis that drives lipid metabolic reprogramming in ccRCC through phosphorylation-dependent regulation.

### CDK13 phosphorylates METTL16 at Ser329 to drive lipid metabolic reprogramming in ccRCC

**Fig. 6:**
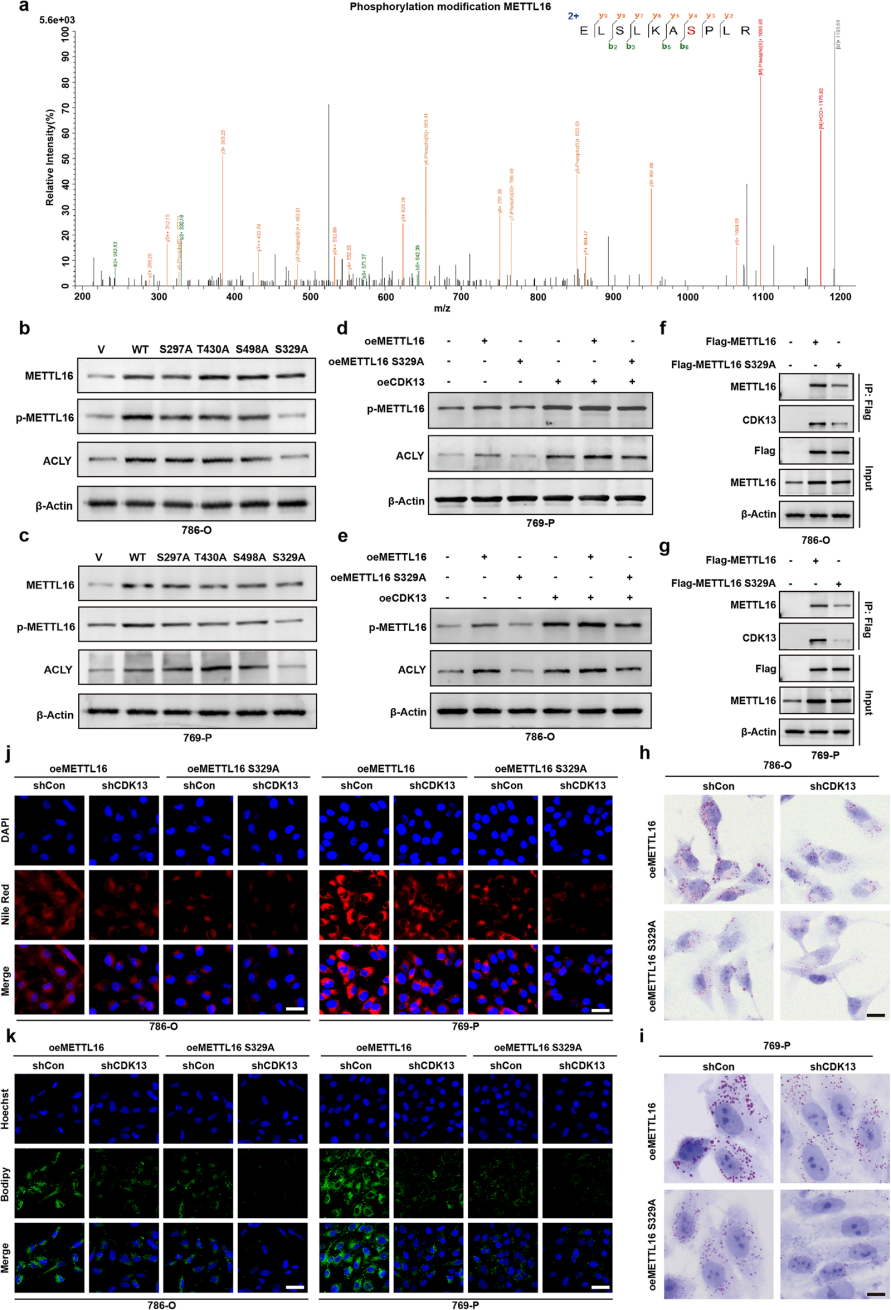
CDK13-mediated phosphorylation of METTL16 drives lipid metabolic reprogramming. **a** Mass spectrometry identification of METTL16 phosphorylation at serine 329 (S329). **b**, **c** Western blot analysis of METTL16, phosphorylated METTL16 (p-METTL16-S329) and ACLY protein levels in 786-O and 769-P cells transfected with WT METTL16 or phosphorylation-deficient mutants (S329A and S329D). **b** Results in 786-O cells. **c** Results in 769-P cells. **d**, **e** p-METTL16-S329 and ACLY expression in 786-O and 769-P cells cotransfected with CDK13-overexpressing (oeCDK13), METTL16-overexpressing (oeMETTL16) or phosphorylation-defective METTL16-S329A vectors versus controls. oeCon, empty vector. **d** Results in 786-O cells. **e** Results in 769-P cells. **f**, **g** Co-immunoprecipitation (Co-IP) analysis of CDK13–METTL16 interaction in cells expressing Flag-tagged METTL16-WT or METTL16-S329A. **f** Results in 786-O cells. **g** Results in 769-P cells. **h**, **i** ORO staining of lipid accumulation in 786-O and 769-P cells with METTL16–CDK13 modulation (oeMETTL16, oeMETTL16-S329A and shCDK13). Scale bar, 25 μm. **h** Results in 786-O cells. **i** Results in 769-P cells. **j**, **k** Lipid droplet visualization via BODIPY 493/503 (green) and Nile Red (red) staining in METTL16–CDK13-manipulated cells. **j** Nile Red staining (red). **k** BODIPY 493/503 staining (green). Scale bar, 50 µm (applies to both). The data represent mean ± s.e.m. of three independent experiments (*P < 0.05, **P < 0.01, ***P < 0.001 versus their corresponding controls).

Given that CDK13 phosphorylates METTL16, we sought to determine whether CDK13 promotes lipid metabolic reprogramming through phosphorylating METTL16. Through mass spectrometry-based phosphoproteomic analysis, we identified Ser329 as the CDK13 phosphorylation site on METTL16 (Fig. 6a). Subsequent prediction of potential phosphorylation sites (Ser297, Thr430 and Ser498) using GPS 6.0 led to the construction of alanine substitution mutants. Functional characterization revealed that while T430A, S498A and S329A mutations globally reduced METTL16 phosphorylation levels, only the S329A mutant abolished METTL16’s ability to upregulate ACLY expression in both 786-O and 769-P cells (Fig. 6b,c), identifying Ser329 as the critical phosphorylation site. Gain-of-function studies with wild-type (WT) METTL16 and the S329A mutant demonstrated that WT METTL16 or CDK13 overexpression alone potently increased p-METTL16 levels and ACLY expression, while co-expression produced additive effects. Conversely, the S329A mutant completely abolished METTL16 phosphorylation, severely reduced ACLY upregulation and weakened CDK13–METTL16 interaction (Fig. 6d–g). Lipid deposition analysis further revealed that S329A mutant-overexpressing cells exhibited markedly reduced lipid accumulation compared with WT METTL16-expressing cells, and CDK13 knockdown partially reversed METTL16-induced lipid promotion (Fig. 6h,i). These findings were corroborated by BODIPY 493/503 and Nile Red staining (Fig. 6j,k). Collectively, these data establish a CDK13–METTL16(Ser329)–ACLY axis that drives de novo lipogenesis and lipid metabolic reprogramming in ccRCC.

### METTL16/YTHDC2-mediated m^6^A modification enhances ACLY mRNA stability

**Fig. 7:**
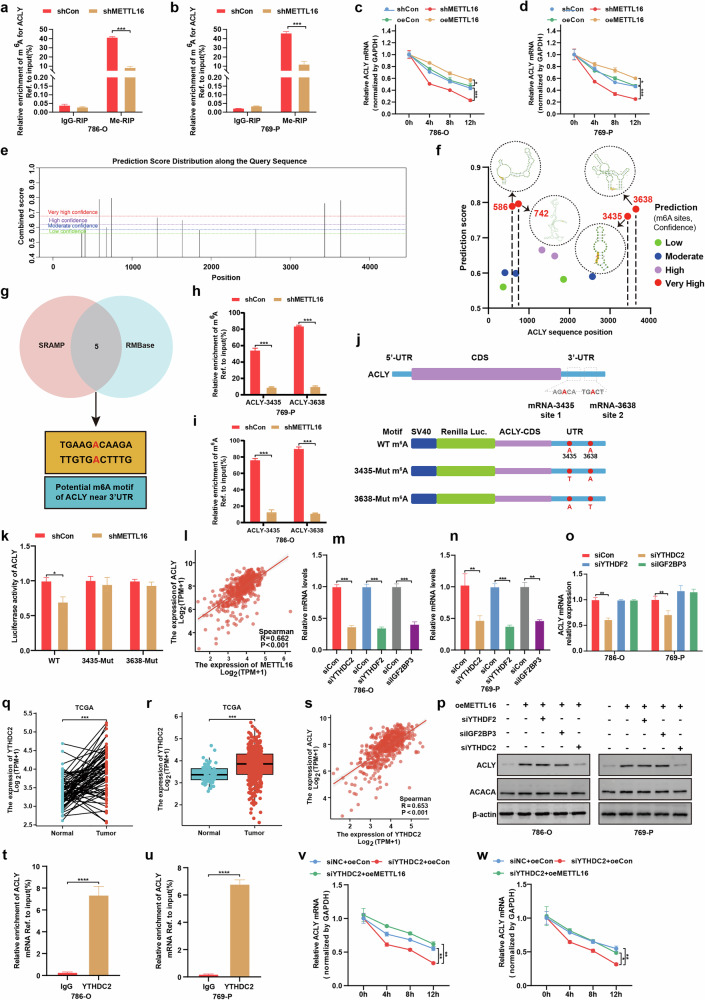
METTL16 stabilizes ACLY mRNA via YTHDC2-dependent m^6^A modification. **a**, **b** Gene-specific m^6^A MeRIP–qPCR analysis demonstrating reduced m^6^A enrichment on ACLY mRNA in METTL16-knockdown (shMETTL16) 786-O and 769-P cells versus scramble controls (shCon). IgG-RIP served as negative control. **a** Results in 786-O cells. **b** Results in 769-P cells. **c**, **d** ACLY mRNA decay rates measured by RT–qPCR in METTL16-modulated cells treated with actinomycin D (5 μg/mL). The data are normalized to GAPDH. **c** Results in 786-O cells. **d** Results in 769-P cells. **e** ACLY m^6^A modification sites predicted using SRAMP (http://www.cuilab.cn/sramp). **f** Secondary structure of high-confidence m^6^A sites predicted by SRAMP. **g** Consensus m^6^A motifs in ACLY 3′UTR identified via SRAMP and RMBase (http://rna.sysu.edu.cn/rmbase/). **h**, **i** MeRIP–qPCR validation of m^6^A peaks at ACLY-3435 and ACLY-3638 loci in METTL16-silenced cells. **h** Results in 769-P cells. **i** Results in 786-O cells. **j** A schematic of ACLY m^6^A motifs in coding sequence (CDS) and 3′UTR. Luciferase reporter constructs (WT and Mut: A-to-T substitutions) are illustrated. **k** Relative luciferase activity of WT/Mut ACLY reporters in shMETTL16 versus shCon 786-O cells. **l** Positive correlation between METTL16 and ACLY mRNA in TCGA ccRCC cohort (Pearson’s correlation). **m**, **n** RT–qPCR confirmation of YTHDC2, YTHDF2 and IGF2BP3 knockdown efficiency in siRNA-transfected cells. **m** Results in 786-O cells. **n** Results in 769-P cells. **o** ACLY mRNA levels in cells transfected with YTHDC2-, YTHDF2- or IGF2BP3-targeting siRNAs. **p** Western blot analysis of ACLY protein in METTL16-overexpressing (oeMETTL16) cells with or without YTHDC2/YTHDF2/IGF2BP3 knockdown. **q**, **r** YTHDC2 mRNA expression in TCGA-KIRC tumors versus normal tissues. **q** Paired analysis. **r** Unpaired analysis. **s** Positive correlation between YTHDC2 and ACLY mRNA in TCGA ccRCC. **t**, **u** RIP–qPCR validation of YTHDC2 protein binding to ACLY mRNA. **t** Results in 786-O cells. **u** Results in 769-P cells. **v**, **w** ACLY mRNA decay rates in METTL16-overexpressing cells with or without YTHDC2 knockdown following actinomycin D treatment. **v** Results in 786-O cells. **w** Results in 769-P cells. The data represent mean ± s.e.m. of three independent experiments (*P < 0.05, **P < 0.01, ***P < 0.001 versus their corresponding controls).

Given the established role of METTL family proteins in m^6^A modification^31^, we explored whether METTL16 regulates ACLY expression through mRNA methylation. MeRIP–qPCR analysis revealed that METTL16 knockdown directly reduced m^6^A levels on ACLY mRNA in both 786-O and 769-P cells (Fig. 7a,b). To determine the functional consequence of this modification, we performed actinomycin D chase assays. METTL16 depletion accelerated ACLY mRNA degradation, while its overexpression stabilized ACLY transcripts (Fig. 7c,d), establishing a direct link between METTL16-mediated m^6^A modification and ACLY mRNA stability. Through SRAMP and RMBase database analysis, we identified two high-confidence m^6^A sites (3435A and 3638A) in the ACLY 3′UTR (Fig. 7e–g). MeRIP–qPCR confirmed that these sites were specifically methylated by METTL16 in both cell lines (Fig. 7h,i). Luciferase reporter assays further validated the functional significance of these m^6^A marks: mutation of both sites (ACLY-3435/3638-Mut) completely abolished METTL16-dependent ACLY promoter activity, while WT ACLY constructs remained responsive to METTL16 (Fig. 7j,k). Besides, analysis of METTL16 and ACLY expression in TCGA database suggested that METTL16 and ACLY were positively correlated in ccRCC (Fig. 7l).

To identify the m^6^A ‘reader’ involved in this process, we focused on YTHDC2 due to its established role in stabilizing m^6^A-modified transcripts^32^. Targeted knockdown of YTHDC2, YTHDF2 and IGF2BP3 revealed that only YTHDC2 depletion specifically reduced ACLY mRNA and protein levels in both cell lines (Fig. 7m–p). This was paralleled by increased YTHDC2 expression in KIRC tissues (Fig. 7q,r), which correlated with ACLY levels (Fig. 7s). RIP assays confirmed direct binding of YTHDC2 to ACLY mRNA (Fig. 7t,u). Finally, we demonstrated that YTHDC2 knockdown attenuated METTL16-induced ACLY mRNA stabilization in both cell lines (Fig. 7v,w). These findings establish a METTL16–YTHDC2 axis that stabilizes ACLY mRNA through 3′UTR m^6^A modification, thereby promoting lipid metabolic reprogramming in ccRCC.

### Targeting the CDK13–METTL16–ACLY axis modulates lipid metabolic reprogramming and suppresses ccRCC progression

**Fig. 8:**
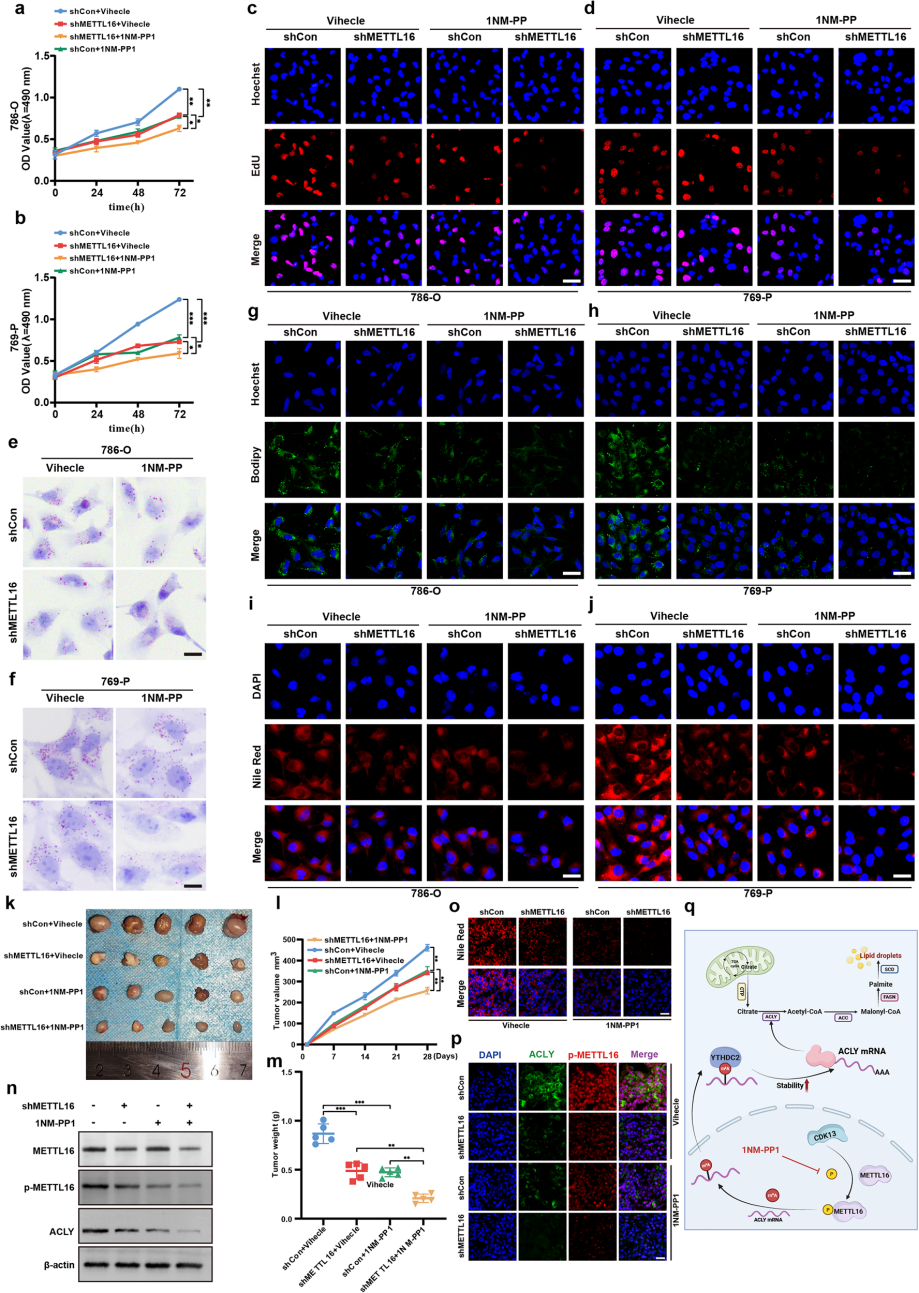
Blockade of CDK13–METTL16–ACLY pathway-mediated lipid metabolic reprogramming inhibits ccRCC progression. **a**–**d** 786-O and 769-P cells were transfected with shMETTL16 and then treated with or without 1NM-PP1 (10 μM), and cell proliferation was assessed by CCK-8 assay and EdU assay. Scale bar, 50 μm. **e–j** 786-O and 769-P cells were transfected as in (**a**) and lipid deposition were measured by ORO staining (**e**, **f**) (scale bar, 25 μm), BODIPY 493/503 (**g**, **h**) and Nile Red staining (**i**, **j**). Scale bar, 50 μm. **k** Xenograft tumor growth in nude mice implanted with METTL16-knockdown 786-O cells. Mice received intraperitoneal 1NM-PP1 (20 mg/kg) or vehicle control every 2 days. Representative tumor images (*n* = 5/group). **l** Tumor volumes were measured with calipers and calculated by the formula: (length × width^2^)/2. **m** Wet weight of the xenograft tumors was determined after tumor resection. **n** Western blot analysis of METTL16, phosphorylated METTL16 (p-METTL16) and ACLY in xenograft tumors. **o** Nile Red staining of lipid deposition (red) with DAPI nuclear counterstain (blue) in tumors. Scale bar, 100 μm. **p** Double immunofluorescence staining of ACLY (green) and p-METTL16 (red) with DAPI (blue) in tumor sections. Scale bar, 100 μm. **q** A schematic of the CDK13–METTL16–ACLY axis governing fatty acid synthesis and lipid deposition via ACLY-driven de novo lipogenesis. The data represent mean ± s.e.m. of three independent experiments (*P < 0.05, **P < 0.01, ***P < 0.001 versus their corresponding controls).

To evaluate the therapeutic potential of disrupting this axis, we investigated the effects of 1NM-PP1, a selective Src family kinase inhibitor (including CDK13)^21^. Building on our discovery that CDK13 phosphorylates METTL16 to promote lipid metabolic reprogramming, we hypothesized that 1NM-PP1 could block this axis. In vitro validation showed that 1NM-PP1 or METTL16 knockdown potently inhibited proliferation in 786-O and 769-P cells (Fig. 8a–d), with combination therapy (1NM-PP1 + METTL16 shRNA) exhibiting synergistic antiproliferative effects. Lipid metabolic analysis revealed that both monotherapy and combination treatment markedly reduced lipid accumulation in vitro (Fig. 8e–j), as assessed by ORO, BODIPY 493/503 and Nile Red staining.

In vivo translation confirmed that 1NM-PP1 or METTL16 knockdown suppressed tumor growth in a 786-O xenograft model, with combination therapy achieving maximal growth inhibition (Fig. 8k). Quantitative analysis showed substantial reductions in tumor volume and weight (Fig. 8l,m), while molecular interrogation revealed that 1NM-PP1 treatment attenuated METTL16 phosphorylation (p-METTL16) and downregulated ACLY expression in tumor tissues (Fig. 8n). Immunofluorescence and Nile Red staining corroborated these findings at both molecular and histopathological levels (Fig. 8o,p). Collectively, our findings reveal previously unrecognized roles of the CDK13–METTL16–ACLY signaling axis in ccRCC pathogenesis. These results suggest that therapeutic modulation of this pathway may offer new treatment opportunities for patients with ccRCC (Fig. 8q).

## Discussion

In this study, we addressed the critical gap in understanding how CDK13 contributes to the metabolic reprogramming characteristic of ccRCC. Although ccRCC is defined by aberrant lipid accumulation^33^, the molecular drivers linking kinase signaling to post-transcriptional RNA modification remained unclear. We now identify CDK13 as a central orchestrator of lipid metabolism in ccRCC, acting through a phosphorylation-dependent epitranscriptomic axis. Specifically, CDK13 phosphorylates the RNA methyltransferase METTL16, enhancing its m^6^A deposition on ACLY mRNA. These m^6^A marks are recognized by YTHDC2, leading to mRNA stabilization, increased ACLY expression, and consequently, enhanced lipogenesis and tumor progression. This CDK13–METTL16–ACLY axis not only clarifies lipid dysregulation in ccRCC but also reveals new crosstalk between kinase signaling and RNA methylation in cancer metabolism.

Our findings extend the known roles of CDK13 and METTL16 in cancer. While CDK13 has been linked to transcriptional regulation and splicing^34,35^, its function in metabolic reprogramming—especially in a lipid-dependent cancer like ccRCC—was unclear. By connecting CDK13 to ACLY stabilization via METTL16, we redefine CDK13 as a key regulator of epitranscriptomic remodeling beyond canonical cell cycle roles. Furthermore, although METTL3 and METTL14 are well-studied m^6^A writers^36^, our work establishes METTL16 as a context-dependent regulator in ccRCC^37^. Unlike METTL3/METTL14, METTL16 is activated by CDK13-mediated phosphorylation, introducing kinase-dependent control as a new regulatory layer for m^6^A writers. This mechanism differs from other m^6^A-mediated metabolic pathways (for example, in hepatocellular carcinoma^38^), underscoring the tissue-specific nature of epitranscriptomic regulation. The positive correlation among CDK13, METTL16 and ACLY in patient cohorts further supports the clinical relevance of this axis.

Critical analysis highlights both the strengths and specificities of our model. Consistency across in vitro, patient-derived, and in vivo models strongly supports the pathway. Identifying Ser329 as the essential phosphorylation site on METTL16 provides key mechanistic insight, yet raises important questions: Why does CDK13 preferentially phosphorylate METTL16 in ccRCC lipid metabolism? Is this due to unique complex formation or cellular localization? Although the pathway operates independently of METTL3/METTL14, interplay with other RNA-modifying enzymes in shaping the m^6^A landscape remains an open area^39^. The synergistic effect of dual CDK13 and ACLY knockdown in vivo indicates a potent functional interaction but also suggests potential compensatory mechanisms—a common challenge in targeted therapy.

The CDK13–METTL16–ACLY axis represents a promising therapeutic vulnerability. Synergistic tumor suppression with CDK13 inhibition and METTL16 knockdown supports combinatorial targeting to overcome resistance. In addition, ACLY expression may serve as a predictive biomarker for patient stratification^40^ and m^6^A patterns on ACLY mRNA in liquid biopsies could enable non-invasive diagnosis or monitoring^39^. Future directions include: (1) elucidating CDK13–METTL16 structural interactions to guide inhibitor design; (2) investigating crosstalk with other ccRCC pathways, such as VHL/HIF^41^ or involved in fatty acid oxidation^42^; and (3) developing and screening for small-molecule modulators that can specifically inhibit the methyltransferase activity of METTL16 in a context-dependent manner^43^.

We demonstrate that CDK13 drives ccRCC progression by rewiring lipid metabolism through a phosphorylation-dependent epitranscriptomic pathway. CDK13 activates METTL16, which deposits m^6^A marks on ACLY mRNA, leading to YTHDC2-mediated stabilization and elevated lipogenesis. This work not only resolves key aspects of lipid dysregulation in ccRCC but also expands the functional scope of CDK13 and establishes METTL16 as a kinase-regulated m^6^A writer with oncogenic roles^37,43^. Targeting this axis holds substantial promise for novel therapies against this metabolically driven malignancy.

## Supplementary information

Supplementary Information

## Data Availability

All data are included within the Article and its Supplementary Information. In this study, the pan-cancer datasets and the KIRC datasets are available in the TCGA database (https://portal.gdc.cancer.gov). Some ccRCC datasets are available in the CPTAC database (https://proteomics.cancer.gov/data-portal/).
