# Supplementary material for: CDK13 drives clear cell renal carcinoma through METTL16-mediated m6A modification of ACLY mRNA
Source: Exp Mol Med. 2026 Feb 12;58(2):472–86. doi: 10.1038/s12276-025-01634-7 (PMC12992896; doi:10.1038/s12276-025-01634-7)
Supplement: Supplementary file 1 — Supplementary Information [file 12276_2025_1634_MOESM1_ESM.pdf]

CDK13 Drives Clear Cell Renal Carcinoma through METTL16-Mediated m^6A Modification of ACLY mRNA

Jinsuo Chen[1,9], Huan Liu[2,9], Yong Zhang[3,9], Junfei Gu[4], Xiaoli Wu[5], Fan Xuan[5], Changbao Qu[6], Hao Sun[7], Nanxi Yue[7], Chenxiao Yang[8], Hongyue Zhao[7], Wenzeng Yang[1]✉ and Zhan Yang[8]✉

[1]School of Clinical Medicine, Hebei University, Department of Urology, Affiliated Hospital of Hebei University, Baoding, 071002, China. [2]Department of Radiotherapy, The First Central Hospital of Baoding, Baoding, Hebei 071000, P.R. China. [3]Department of Urology, National Cancer Center/National Clinical Research Center for Cancer/Cancer Hospital, Chinese Academy of Medical Sciences and Peking Union Medical College, Beijing 100021, China. [4]Department of Urology, The Second Hospital of Hebei Medical University, Shijiazhuang 050000, China. [5]Department of Pediatrics Hematology-Oncology, the Second Hospital of Hebei Medical University, Shijiazhuang 050000, China. [6]Department of Urology, Shijiazhuang People's Hospital, No. 365, Jianhua South Street, Yuhua District, Shijiazhuang, Hebei, China. [7]Department of Biochemistry and Molecular Biology, The Key Laboratory of Neural and Vascular Biology, Ministry of Education of China, Hebei Medical University, Shijiazhuang, Hebei 050017, P.R. China. [8]Center of Tumor Immunology and Cytotherapy, Medical Research Center, The Affiliated Hospital of Qingdao University,

Qingdao, 266000, Shandong, China. [9]These authors contributed equally: Jinsuo Chen, Huan Liu and Yong Zhang. ✉email: bdywz@sina.com; yangzhan@hebmu.edu.cn

Supplementary Information

Supplementary Figures

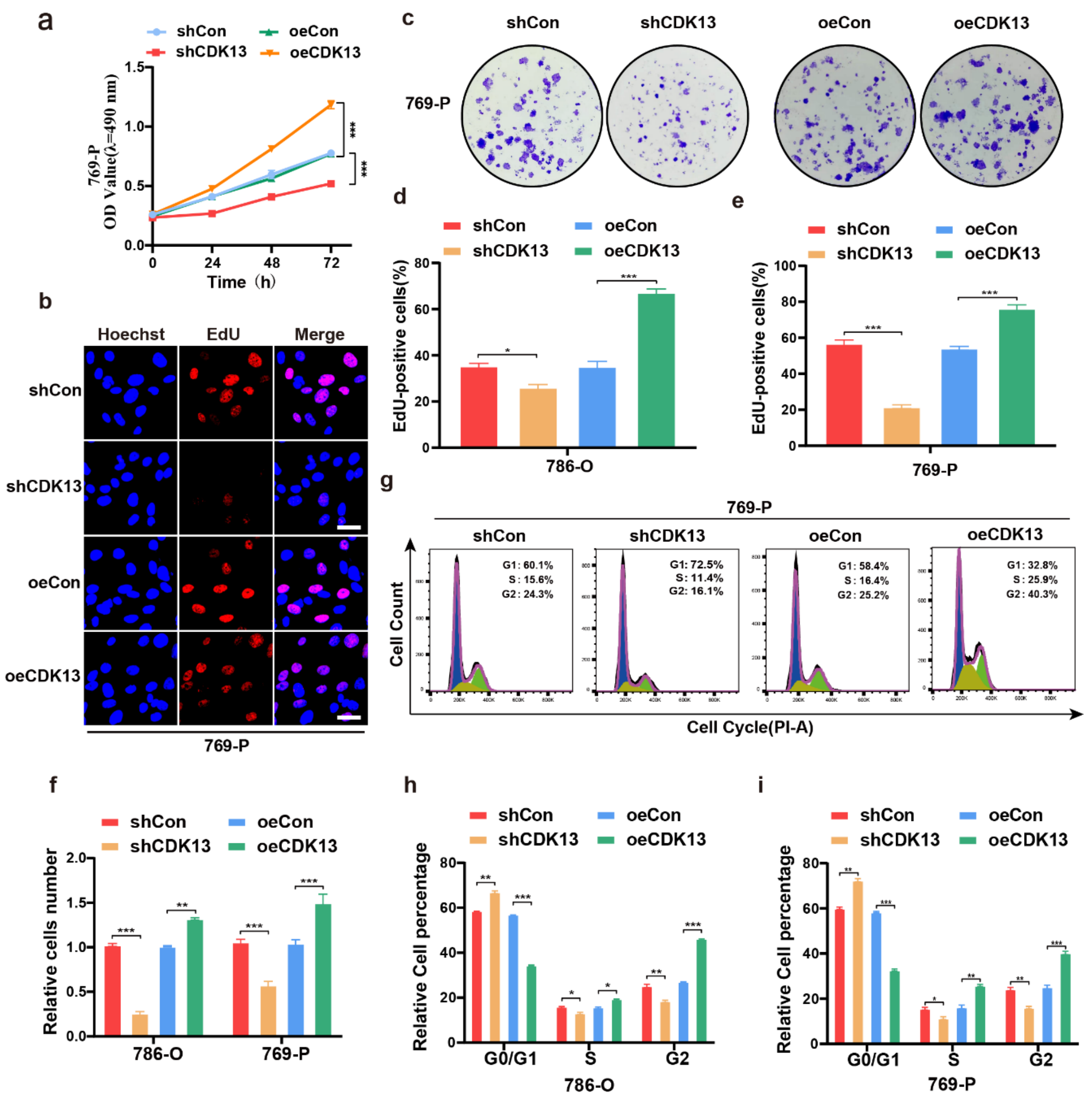

Supplementary Fig. 1 CDK13 enhances ccRCC cell proliferation in vitro

(a) Cell viability assessed by CCK-8 assay and colony formation assay (c) in 769-P

cell transfected with CDK13-overexpressing (oeCDK13) or CDK13-knockdown (shCDK13) vectors versus their respective controls (oeCon: empty overexpression vector; shCon: scramble shRNA control). (b) EdU incorporation assay evaluating proliferation in 769-P cell with CDK13 modulation. Scale bar = 50 μm. (d) Quantitative analysis of EdU-positive cells in (Fig. 2b). (e) Quantitative analysis of EdU-positive cells in (b). (f) Quantification of colony formation results from (Fig. 2c) and (c). (g) Cell cycle phase distribution analyzed by flow cytometry in CDK13-manipulated 769-P cell. (h) Histograms showing percentage of 786-O cell (Fig. 2d) in G0/G1, S, and G2/M phases under different CDK13 expression conditions. (i) Histograms showing percentage of 769-P cell (g) in G0/G1, S, and G2/M phases under different CDK13 expression conditions. (*P < 0.05, **P < 0.01, ***P < 0.001 vs. corresponding controls).

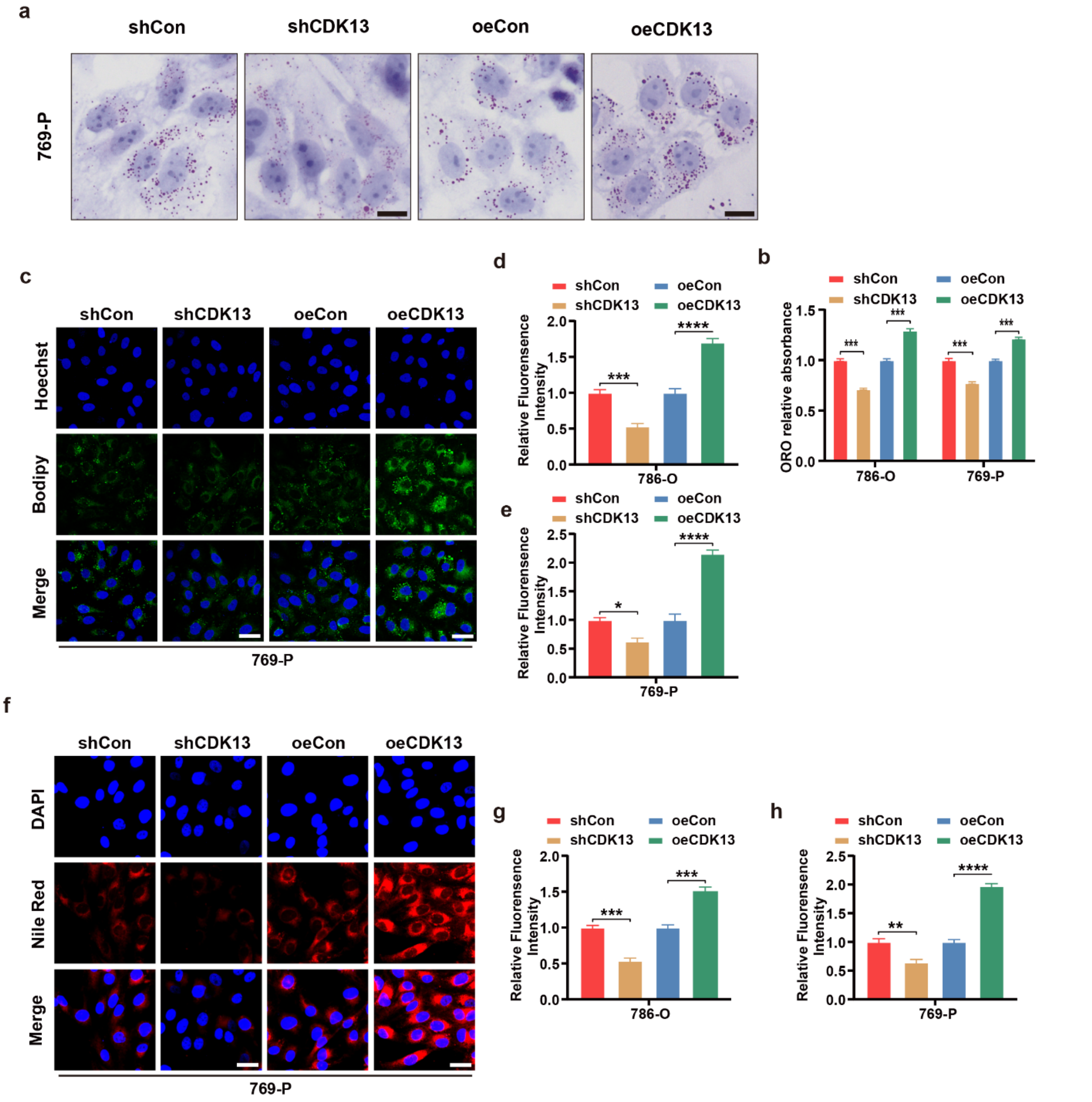

Supplementary Fig. 2 CDK13 drives fatty acid biosynthesis and lipid accumulation in ccRCC

(a) ORO staining of lipid accumulation in 769-P cell transfected with CDK13-overexpressing (oeCDK13), CDK13-knockdown (shCDK13) vectors, or their respective controls (oeCon: empty vector; shCon: scramble shRNA). Scale bar = 25

μm. (b) Quantification of ORO staining intensity in (Fig. 2h) and (a). (c) BODIPY 493/503 staining of lipid droplets (LDs) in 769-P cell visualized by confocal microscopy. Scale bar = 50 μm. (d, e)Quantification of BODIPY 493/503 fluorescence intensity in (Fig. 2i) and (c). (f) Nile Red staining of neutral lipids in CDK13-modulated 769-P cell. Scale bar = 20 μm. (g, h) Quantitative analysis of Nile Red fluorescence intensity in (Fig. 2j) and (f). (*P < 0.05, **P < 0.01, ***P < 0.001 vs. corresponding controls).

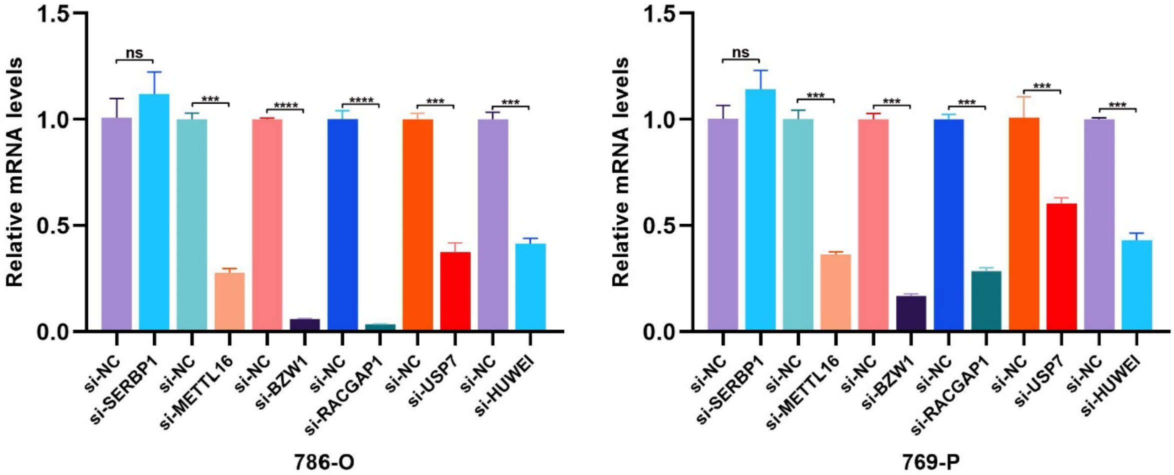

Supplementary Fig. 3 786-O and 769-P cells were transfected with the specified siRNA, and then the mRNA expression of these genes was detected by qRT-PCR. (**P* < 0.05, ***P* < 0.01, ****P* < 0.001 vs. corresponding controls).

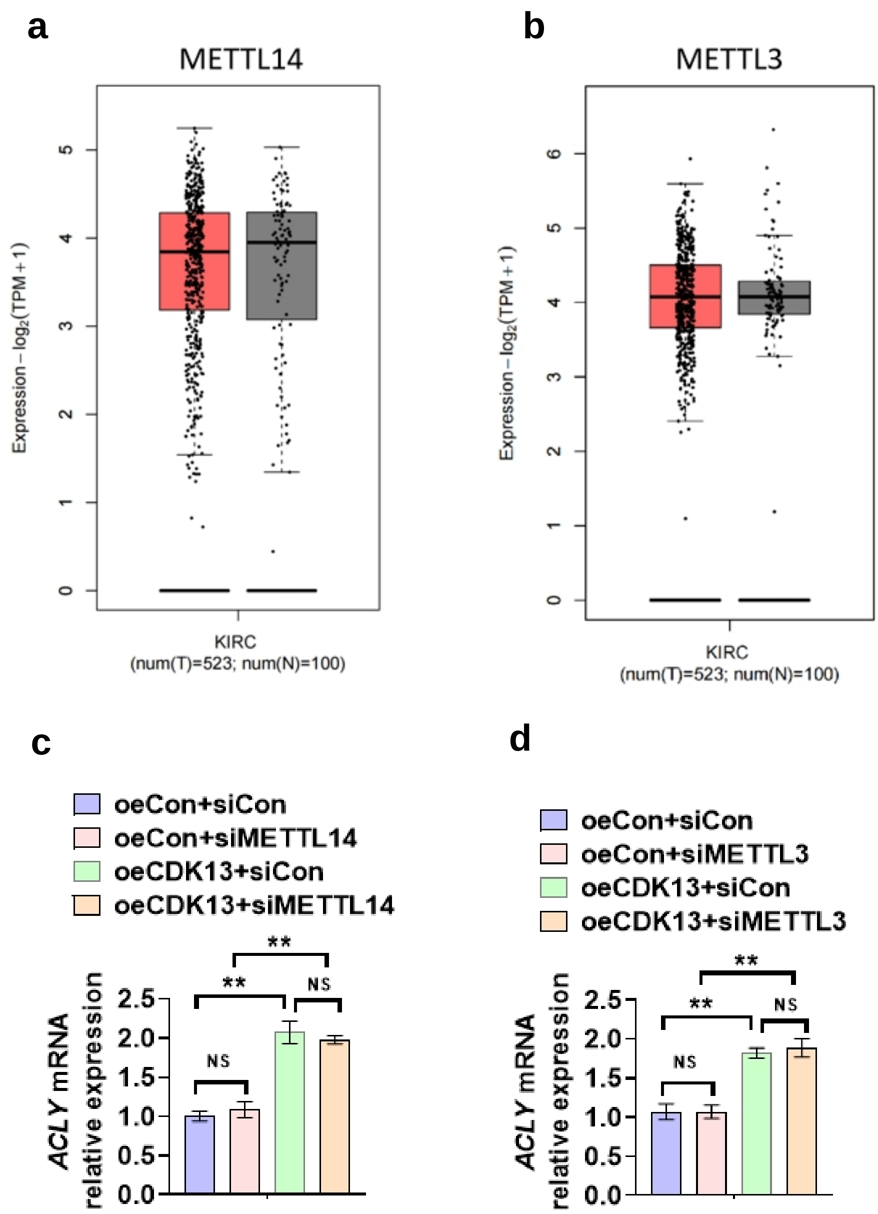

Supplementary Fig. 4 (a,b) METTL14 and METTL3 expression in ccRCC from the TCGA database. (c) 786-O cells were transfected with oeCDK13 or siMETTL14 or both together, and then RT-qPCR detected ACLY expression. (d) 786-O cells were transfected with oeCDK13 or siMETTL3 or both together, and then RT-qPCR detected

ACLY expression. (*$P < 0.05$, **$P < 0.01$, ***$P < 0.001$ vs. their corresponding controls).

Supplementary Table I The top 34 significantly different genes

	shCDK13-1	shCDK13-2	shCDK13-3	shCon-1	shCon-2	shCon-3
ACLY	11.51393319	11.1299731	10.31158608	44.52984	44.03475	39.70321
FASN	95.46103002	81.839416	100.9238804	318.4409	239.9126	205.1247
ACACA	1.959930292	1.75048271	2.18977936	3.55557	3.045869	3.468041
CDK13	0.039005673	0.091005	0.109879296	0.172663	0.179058	0.159974
PHB2	94.84739235	117.665285	118.3761632	176.8723	212.8367	244.1805
RACK1	686.6529022	504.375541	489.7739664	1640.419	1227.905	1070.316
ENO1	1773.730191	1859.95219	1827.787042	2381.462	2758.862	2804.558
EIF5A	264.1017462	263.895222	271.4597815	556.7689	627.2241	614.6761
FADS2	3.714837406	3.58544592	3.1720355	5.661797	5.153936	5.263514
USF1	11.73691183	13.1375821	15.86617022	20.77655	23.32107	21.03871
AHSA1	29.78438325	32.5777156	35.67383403	78.50507	68.81646	67.1278
FUS	38.58943424	37.1996822	40.66488886	78.07833	86.94648	80.87769
RAN	30.85187666	43.2648372	40.23922436	77.89518	100.5866	99.57487
STIP1	16.16897594	22.7851941	23.61340504	72.39948	67.31624	74.93651
RPP25L	35.66633387	46.4380927	37.49623858	71.47277	58.26607	59.39956
CDK4	50.48186332	54.8820788	61.22423095	71.41585	74.77998	71.76571
TMEM161A	126.3013748	92.3011069	86.48282271	61.82671	66.37139	62.54267
SNU13	44.65979224	45.0378189	40.41770909	61.25551	67.76427	65.1012
NRBP1	28.19526151	28.7947006	33.58130182	60.69732	69.40631	71.56748
RCC1L	43.96269656	49.9349645	47.70300128	60.20882	66.28809	62.47659
DUS3L	76.95051897	75.4930189	70.31402096	60.02058	62.04791	55.83364
SNAPC2	120.5371216	106.935563	116.8889549	76.72264	70.28723	83.35152
UBTD1	88.80386455	62.7882371	63.50918437	52.22902	47.55508	45.39902
N4BP3	2.431953524	2.31727694	2.136921304	0.714226	0.853044	1.25683
POLR2L	199.8286508	247.413084	218.0392754	622.493	536.9709	525.2023
PSMB5	165.0756897	205.332578	195.2938745	282.69	339.5429	348.0279
DPP7	784.769335	693.161691	629.6292067	371.3837	329.5281	311.0407
PKN1	647.4691992	593.37275	564.2929774	332.4574	361.3054	366.1725
UBE2M	315.948701	385.318699	354.5456272	244.7779	256.832	262.3189
CPNE7	155.4761164	125.383195	130.4194837	88.79526	84.45198	76.62153
POLD1	152.3392351	127.432912	115.3394171	78.406	68.44919	60.3733
ZNF787	144.501053	131.27576	126.8492143	60.0241	74.37094	71.53212
DDIT4	138.8630356	105.359981	100.1584571	86.21762	80.18793	69.25471
STUB1	130.3920163	117.975111	110.0114111	79.2863	89.38716	80.25553

Supplementary Table II The top 42 significantly different proteins

label	shCDK13-1	shCDK13-2	shCDK13-3	shCon-1	shCon-2	shCon-3
METTL16	27329	24673	26388	32993	34176	35900
HUWE1	185390	172280	176500	272240	265520	280000
USP7	35137	30561	25103	65284	60518	68080
ELF4	85870	88356	97688	142860	135310	133850
CLASP1	43071	49745	45908	79354	76655	72408
PHF2	44943	41987	35682	57977	63767	60852
RPL13	85511	70952	69394	116650	101680	120707
YIF1A	46478	29463	38670	105340	91583	99063
PRKCA	8456800	6886000	7458400	12000000	9466800	10046000
PRPF4B	25305	26688	21982	35686	33727	36242
IRF2BP1	138910	140790	152740	199730	183460	212760
ATG4B	50717	56474	58558	77406	79465	83433
TMEM87B	341400	317680	357440	736440	602910	603040
TFEB	207270	208780	240590	420600	422210	461460
MAP4	104700	137270	128030	191120	184970	245330
NIFK	158750	122390	130580	101170	87678	110040
PPIG	417150	393620	463070	263350	291670	103540
RFX5	117356	106410	121710	59763	62178	70383
MPZL1	164330	179730	181070	79643	86517	79636
PML	1439000	1555300	1467200	750370	699050	767300
EPHB2	956040	726010	808510	361500	461820	486050
DST	64514	54878	67382	30907	43194	35380
ABLIM3	652500	499310	522360	279810	297520	261690
SORBS1	306270	319480	298900	88499	82198	97230
NDC1	283450	257530	254900	112330	159580	164460
STT3B	1076310	1249000	1141600	525490	551440	705770
POGZ	190030	185410	183680	98920	97939	108780
PHF14	74877	68548	64818	36634	36645	35896
BCKDK	251960	259220	263440	205140	191110	193480
PNKP	687220	581640	607200	471740	473850	472840
HRH1	96877	92496	93532	60962	59648	73773
TEX264	75392	62372	74689	43340	52988	46280
CHD2	105490	98098	108530	58207	70979	65280
CDC6	15880	14960	15724	32130	30286	29274
COBLL1	21850	22160	23070	107225	120852	148700
DNAJC2	18355	22446	19647	33204	33166	37653
SH3BP5L	10013	9594	10233	17662	18773	18542

WDR44	23381	20287	22433	40826	37349	41496
CAV2	576340	548080	530270	241350	279230	261910
NAV3	40873	36489	44399	19805	16877	19549
EIF4G1	1477100	1348200	1347200	1819440	2240300	2142500
LIG1	285960	291840	257530	439100	421670	529190

Supplementary Table III Oligonucleotides were used in this study

Name	Sequcence 5’ to 3’
CDK13-F:	CCCTGAGCTACCAGGAGGAGATG
CDK13-R:	CAGTGTCTTTATCCCTGGCTTTGT
ACLY-F:	GATTTTGCGGGGTTCGTCG
ACLY-R:	GACCCCAACGAGACCAAGTT
ACACA-F:	GCTCCTTGTCACCTGCTTCTGTTGGC
ACACA-R:	CGAGTAACAAATTCTGCTGGAGAAGCCA
ACACB-F:	CGCTGAGTTTGTCACACGCTTTGGGG
ACACB-R:	GGTCCTCGGGGGTCACCATCAC
FASN-F:	CGCGTGGCCGGCTACTCCTAC
FASN-R:	CGGCTGCCACACGCTCCTCT
SCD1-F:	GCCCCTCTACTTGGAAGACGA
SCD1-R:	AAGTGATCCCATACAGGGCTC
HUWEI-F:	CAAACTACATCACTCGTCTGGG
HUWEI-R:	AGTCTCTGCAACATTCTGCAAG
USP7-F:	TCGTCGCACATTGAGACGG
USP7-R:	CTTGTCGGCATGGTTGGGAAT
RACGAP1-F:	ACCTCTTCTGACCTTTCGCC
RACGAP1-R:	CTGAGCCACTCTCTGCAAGT
BZW1-F:	TCTTTCCTGCCAATAAGCAA
BZW1-R:	CTTGACCAGACTATTCCGAT
METTL16-F:	CTCTGACGTGTACTCTCCTAAGG
METTL16-R:	TACCAGCCATTCAAGGTTGCT
SERBP1-F:	CTGACCGACATCGAAGGTGA
SERBP1-R:	AAGTGCAATCCATGGCTCCG
YTHDC2-F:	GGTATCCCCTGCCGTATTTTTG
YTHDC2-R:	CTTTCCCGTCTCTCTGCGG
YTHDF2-F:	AGCCCCACTTCCTACCAGATG
YTHDF2-R:	TGAGAACTGTTATTTCCCCATG
IGF2BP3-F:	GCAGTTTCCGAGTCAGTGTTCA
IGF2BP3-R:	ACGAAATATCCCGCCTCATTTAC
ACLY-365-F:	TCTGGATGGGGTCAAGTCCT
ACLY-365-R:	GAAGGGCTCGATCAGAACGT
ACLY-406-F:	TCTGGATGGGGTCAAGTCCT
ACLY-406-R:	CCTTCTCGGGTGGCATAGAT
ACLY-586-F:	TGTGGACGTGGGTGATGTG
ACLY-586-R:	TTCTTGTCTTCAGGGGCGTG
ACLY-676-F:	GCCAGTTTTATCTCCGGCCT

ACLY-676-R:	CCGCCAGGTCAAGGACATAG
ACLY-742-F:	CCAGTTTTATCTCCGGCCTCT
ACLY-742-R:	TCACCCCACTTCACTTTGCA
ACLY-1320-F:	TCCCCATCCATGTCTTTGGC
ACLY-1320-R:	CTGGCGTTGAGGAGGAAGTT
ACLY-1636-F:	CTATGTCTGCTCCCGAGACG
ACLY-1636-R:	CAGGATCTCTTTGTGCCCCC
ACLY-1855-F:	CTCCGCTCTGCCTATGACAG
ACLY-1855-R:	ATCAGCTTTCTCGTGAGGGC
ACLY-2566-F:	TGCAGTAGCCAAGAACCAGG
ACLY-2566-R:	CGGATCAAACCAAGCTCCCT
ACLY-3435-F:	CAGGGGCTGTATCGTCATCC
ACLY-3435-R:	CACTGCCAGCTGTCTGTACA
ACLY-3638-F:	GGCTAACACCCCTTCAGTCC
ACLY-3638-R:	AGGAAGACCCCATCCTGACA
GAPDH-F:	TGACCTGCCGTCTAGAAAAACCT
GAPDH-R:	GCTGTTGAAGTCAGAGGAGACCA
β-Actin-F:	CATGTACGTTGCTATCCAGGC
β-Actin-R:	CTCCTTAATGTCACGCACGAT
si-SERBP1-F:	CGACAAGUCAAGUGCUUCUTT
si-SERBP1-R:	AGAAGCACUUGACUUGUCGTT
si-METTL16-F:	GGUCCAGCAUAAACGAGUUTT
si-METTL16-R:	AACUCGUUUAUGCUGGACCTT
si-BZW1-F:	GGAUAACAGACUGAUGGAATT
si-BZW1-R:	UUCCAUCAGUCUGUUAUCCTT
si-RACGAP1-F:	GGAUACUAUGAUGCUGAAUTT
si-RACGAP1-R:	AUUCAGCAUCAUAGUAUCCTT
si-USP7-F:	GGAGCGACUUGUUACAUGATT
si-USP7-R:	UCAUGUAACAAGUCGCUCCTT
si-HUWEI-F:	GCAGAAUACUAUGGCCCAUTT
si-HUWEI-R:	AUGGGCCAUAGUAUUCUGCTT
si-YTHDC2-F:	GCCCAUAGAAUAGCUAAUATT
si-YTHDC2-R:	UAUUAGCUAUUCUAUGGGCTT
si-YTHDF2-F:	GCCCUACUUAACUUCUUAUTT
si-YTHDF2-R:	AUAAGAAGUUAAGUAGGGCTT
si-IGF2BP3-F:	GGCUCAGGGAAGAAUUUAUTT
si-IGF2BP3-R:	AUAAAUUCUUCCCUGAGCCTT
